# Prevalence, causes and impact of *TP53*-loss phenocopying events in human tumors

**DOI:** 10.1186/s12915-023-01595-1

**Published:** 2023-04-24

**Authors:** Bruno Fito-Lopez, Marina Salvadores, Miguel-Martin Alvarez, Fran Supek

**Affiliations:** 1 Institute for Research in Biomedicine (IRB Barcelona), The Barcelona Institute for Science and Technology (BIST), Barcelona, Spain; 2 Catalan Institution for Research and Advanced Studies (ICREA), Barcelona, Spain

**Keywords:** Tumor evolution, Driver genes, p53 pathway, Transcriptomic signature, Gene expression, CRISPR screens, Drug resistance

## Abstract

**Background:**

*TP53* is a master tumor suppressor gene, mutated in approximately half of all human cancers. Given the many regulatory roles of the corresponding p53 protein, it is possible to infer loss of p53 activity – which may occur due to alterations in *trans* – from gene expression patterns. Several such alterations that phenocopy p53 loss are known, however additional ones may exist, but their identity and prevalence among human tumors are not well characterized.

**Results:**

We perform a large-scale statistical analysis on transcriptomes of ~ 7,000 tumors and ~ 1,000 cell lines, estimating that 12% and 8% of tumors and cancer cell lines, respectively, phenocopy *TP53* loss: they are likely deficient in the activity of the p53 pathway, while not bearing obvious *TP53* inactivating mutations. While some of these cases are explained by amplifications in the known phenocopying genes *MDM2*, *MDM4* and *PPM1D*, many are not. An association analysis of cancer genomic scores jointly with CRISPR/RNAi genetic screening data identified an additional common *TP53*-loss phenocopying gene, *USP28*. Deletions in *USP28* are associated with a *TP53* functional impairment in 2.9–7.6% of breast, bladder, lung, liver and stomach tumors, and have comparable effect size to *MDM4* amplifications. Additionally, in the known copy number alteration (CNA) segment harboring *MDM2*, we identify an additional co-amplified gene (*CNOT2*) that may cooperatively boost the TP53 functional inactivation effect of MDM2. An analysis of cancer cell line drug screens using phenocopy scores suggests that TP53 (in)activity commonly modulates associations between anticancer drug effects and various genetic markers, such as *PIK3CA* and *PTEN* mutations, and should thus be considered as a drug activity modifying factor in precision medicine. As a resource, we provide the drug-genetic marker associations that differ depending on TP53 functional status.

**Conclusions:**

Human tumors that do not bear obvious *TP53* genetic alterations but that phenocopy p53 activity loss are common, and the *USP28* gene deletions are one likely cause.

**Supplementary Information:**

The online version contains supplementary material available at 10.1186/s12915-023-01595-1.

## Background

Mutations in the *TP53* tumor suppressor gene are a very common feature across almost all types of human cancer. These mutations abrogate or reduce *TP53* activity via various mechanisms: dominant-negative acting missense mutations, loss-of-function missense, nonsense, frameshift indel, splice site, or synonymous mutations, or copy number losses that frequently delete one *TP53* allele while the other allele is inactivated by a mutation. That such *TP53* genetic alterations occur at high frequency in many cancer types implies that they have very strong selective advantages for the expanding cancer cell clones [1, 2]; indeed this is borne out in experimental data on cell lines and animal models of cancer [3, 4].

The large selective advantage of *TP53* losses are consistent with its roles in arresting the cell cycle or triggering apoptosis upon threats to genome integrity. *TP53*-null cells better tolerate genomic instability, which can result from endogenous causes, most prominently oncogene-overexpressing and thus replication-stressed cancerous genetic backgrounds. Consistently, *TP53*-mutant tumors have higher frequencies of segmental copy number alterations (CNA), whole-genome duplication, and overall mutation rates [5, 6]. Moreover, *TP53*-null cells better tolerate DNA damaging conditions that would normally trigger cell cycle checkpoints, such as those resulting from DNA-acting drugs or radiation [7, 8]. Consistently, *TP53* mutation-bearing tumors tend to be more resistant to various cancer chemotherapies [4, 9–11] and radiotherapy [10–12], and more aggressive *TP53* R273 and R248 mutants are associated with accelerated cancer progression in colorectal tumors [13].

The frequency of *TP53* mutations – highest of all cancer genes, standing at 37% in The Cancer Genome Atlas (TCGA) cohort – indicates that most cancers benefit from the loss of *TP53*. However, there are nonetheless many tumors which do not bear a mutation in *TP53*. A part of those is explained by genetic events that phenocopy *TP53* loss i.e. that have similar downstream phenotypic consequences as *TP53* gene loss itself. There are three established examples of *TP53* loss phenocopying events occurring in tumors. Most prominently, this is the amplification of the *MDM2* and *MDM4* oncogenes and overexpression of the corresponding proteins. These negatively regulate *TP53* protein levels by promoting its proteasomal degradation, and otherwise inhibit *TP53* activity by binding to its transactivation domain [14–16]. The third implicated gene is *PPM1D*, whose amplification overexpresses a serine/threonine phosphatase acting upon various targets including *TP53*, reducing its activity. (We note that *PPM1D* can also be affected by point mutations that result in gain-of-function [17–19]).

Given the strong selective advantages of the *TP53* activity loss in cancer evolution, we hypothesized that *TP53* loss phenocopying in human cancers extends beyond these known examples of *MDM2*, *MDM4* and *PPM1D* alterations. If indeed other common mechanisms of *TP53* phenocopying exist, this would be relevant to predicting tumor cell response to various drugs, and to predicting tumor aggressiveness, thus having implications to personalized medicine. Because *TP53* loss has clear consequences on the mRNA expression levels of various downstream targets [4, 20], the *TP53*-null-like phenotype can be inferred from large scale transcriptomic data [20–23]. Here, we apply a statistical framework to jointly analyse 966 cancer cell line and ~7000 tumor genomes and transcriptomes, aiming to identify additional *TP53* phenocopying genetic events and impact on drug sensitivity. We find that *TP53* loss phenocopies are remarkably common across tumors and cancer cell lines, and we identify *USP28* gene deletions as one cause of *TP53* loss phenocopying, and reveal many links between drugs and their targets that are modulated by TP53 activity.

## Results

### Inferring the functional *TP53* status of tumors from transcriptomes

We developed a machine learning method to detect *TP53* loss phenocopies in tumors and cell lines, integrating RNA-seq data with *TP53* mutation data in a logistic regression, regularized with an Elastic Net penalty (very similar cross-validation accuracy was obtained with Ridge or Lasso penalties; see Methods). Regression models were trained using cross-validation on mRNA levels of 7131 tumor samples from the TCGA project, across 20 different cancer types, controlling for cancer type. In addition to using this global analysis of mRNA expression levels to infer the functional *TP53* status state of each tumor, we also identified the genes whose expression patterns are associated with *TP53* status. Tumors with *TP53* putatively causal mutations were included as positive examples (*TP53* status was categorized according to Genomics of Drug Sensitivity in Cancer Project (GDSC) methodology; see Methods). Previously known phenocopying events (*MDM2*, *MDM4* and *PPM1D* amplifications), as well as samples with *TP53* deletions were excluded from the training set (these known phenocopying events will be used to calibrate decision thresholds; see below). Our classifier learned a combination of relevant gene weights that differentiate samples with an aberrant *TP53* activity. Tumor samples that are not *TP53* mutated (by GDSC criteria), but are classified as mutated by the machine learning model are considered to be *TP53* loss phenocopies.

**Fig. 1:**
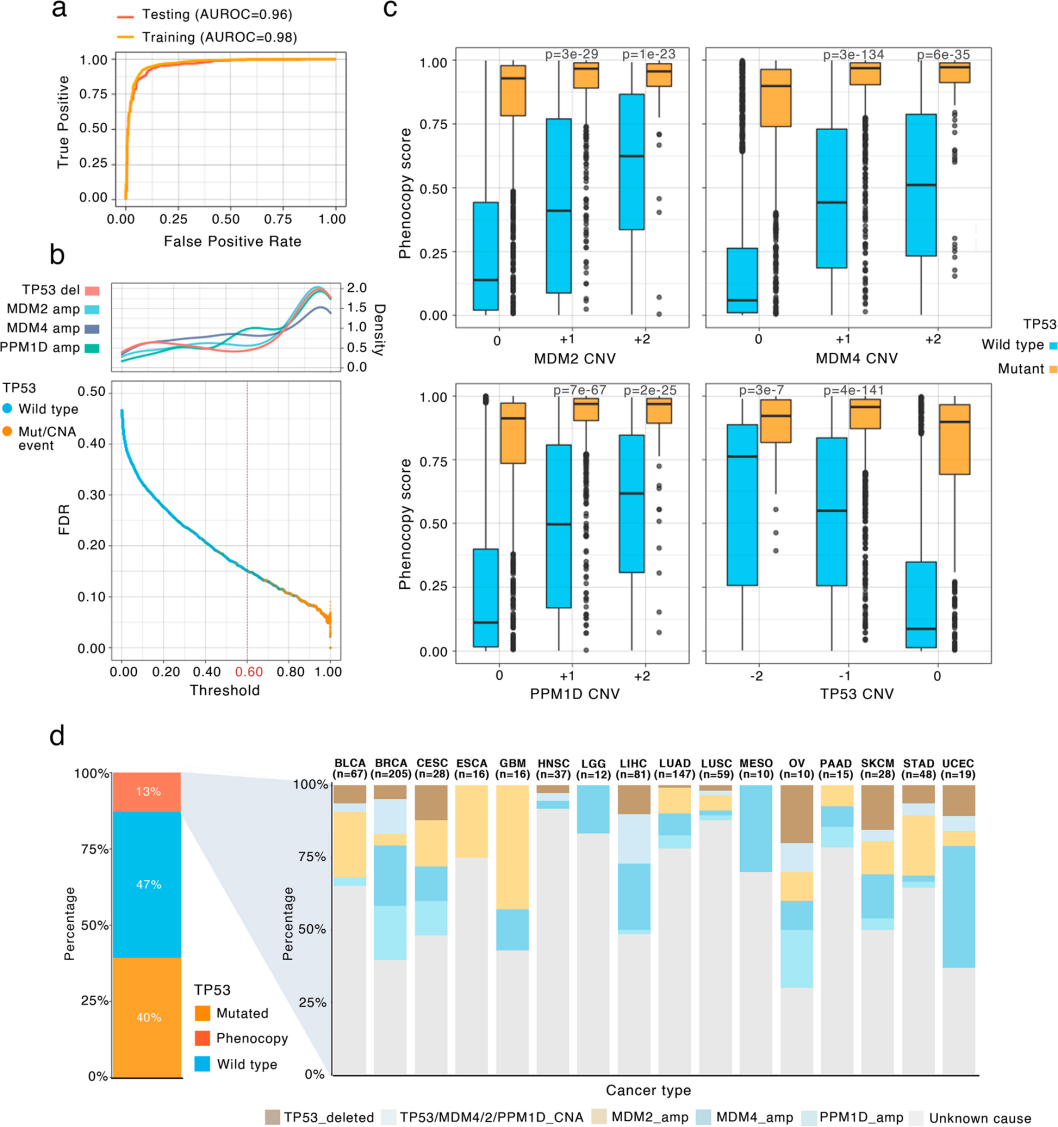
Evaluation of the *TP53* function loss score classifier and prevalence of *TP53* loss phenocopying events in cancer. **a** Receiver operating characteristic (ROC) curve and area under the ROC (AUROC) curve for training and testing sets in TCGA tumor transcriptomes. **b** Bottom: FDR for each tumor sample. X axis shows the classification score thresholds for each tumor sample. The general threshold used for classification (0.6) is highlighted. Top: the histogram of frequency of CNV events (“density” refers to smoothed relative frequency) affecting *TP53* and the known phenocopying genes *MDM4*, *MDM2* and *PPM1D* at various phenocopy score thresholds. **c** *TP53* loss phenocopying score stratified by 3 known phenocopying CNA events and by *TP53* deletions. Data points are tumor samples coloured by *TP53* status; boxes show median, Q1 and Q3, while whiskers show range (outlying examples shown as separate dots). X axis represents the GISTIC thresholded CNV of each given gene. Tumor samples with deletions in the corresponding genes (for *MDM2*, *MDM4* and *PPM1D*) and amplifications (*TP53*) are omitted for simplicity. *P* values are by t-test comparisons of the TP53 phenocopy score between each shown CNV category to the neutral CNV (0) category in *TP53* wild-type samples. **d** *TP53* functional status distribution across TCGA cancer types. Left: pan-cancer; “*Phenocopy*” refers to *TP53*-loss phenocopying tumors according to the classifier in panels **a** and **b**. Right: showing only the* TP53* loss phenocopying tumor samples by cancer type, further stratified by cause of the phenocopy. Tumor samples harbouring a known event that affects *TP53* functionality are shown with colours, and the remaining *TP53*-loss phenocopy tumors are labelled as “Unknown cause”. KIRC and DLBC were omitted as they only had 1 phenocopied tumor sample (”unknown cause”)

Our classifier showed a high performance with an area under the receiver operating characteristic (AUROC) curve of 96% in cross-validation on TCGA tumors (out-of-sample accuracy), and 95% on the testing set (consisting of 10% of the samples held out from training set, Fig. 1a). Thus, we were able to often correctly detect *TP53* status in tumor samples the classifier was not exposed to, with an area under precision-recall curve = 0.9654. The *TP53* loss phenocopy scores for each TCGA tumor sample are provided in Additional file 3: Data S1.

To account for cancer type-specific effects, our classifier included the cancer type as a variable. Leave-one-cancer-type-out (LOCTO) cross-validation showed that classifier performance was robust to removal of individual cancer types, with LOCTO AUCs ranging from 0.951 (LGG removal) to 0.964 (SKCM removal) compared to an are under the curve (AUC) of 0.961 using the full dataset (Additional file 1: Fig. S1a). *TP53* phenocopy scores from the LOCTO models were highly correlated with those from the full model (e.g. R = 0.89, *p* = 4e-163, for BRCA removal, Additional file 1: Fig. S1b), indicating that the classifier would generalize well to unseen cancer types. Additionally, we tested for correlations between tumor purity estimates and TP53 phenocopy scores across all cancer types and found no significant relationship overall (pan-cancer Pearson R = -0.03, *p* = 0.11, 1/10 cancer types was found correlated, Additional file 1: Fig. S1c).

Out of the ~ 12,000 genes available to the classifier, 217 genes were deemed relevant for *TP53* status classification (non-zero coefficients; gene score provided in Additional file 3: Data S2). These represent a sparse (but not necessarily exhaustive) set of genes that are, considered together, highly informative for predicting *TP53* functional status. Expectedly, many of the classifier's most relevant genes are known to be related to TP53 functionality. For instance, *apoptosis-enhancing nuclease (AEN)* was the gene with the highest absolute importance score. This exonuclease is a direct *TP53* target whose expression is regulated by the phosphorylation of *TP53* and its tumor suppressor role has been reported [24]. Tumors with a high expression of *AEN* are expected to be p53 functional, and indeed highly expressed *AEN* was associated with *TP53* WT status in our classifier’s coefficients. On the other extreme, *COP1*, a ubiquitin ligase that acts as an important p53 negative regulator, was the highest negative importance score with *TP53* mutated status in the classifier [25]. We further performed a GO enrichment analysis, revealing that top functional enriched sets were related to apoptotic signals, supporting the biological rationale underlying this set (Additional file 1: Fig. S1d). Most enriched pathways were*: intrinsic apoptotic signalling pathway in response to DNA damage by p53 class mediator* (8.1-fold enrichment, false discovery rate (FDR) = 4.2%), *pyrimidine deoxyribonucleoside monophosphate biosynthetic process* (47.4 fold enrichment, FDR = 1.9%) and *response to UV-B* (17.2 fold enrichment, FDR = 3.7%) (ShinyGO, see Methods).

### Validation and calibration of the TP53 phenocopy score model

Our classifier extends recent gene expression-based models for TP53 functionality [20–23] by being able to generalize across both tumor and cancer cell lines (important for identifying drug sensitivity associations, see below), and moreover it can provide calibrated FDR estimates for TP53 functional status of each tumor or cell line. In particular, to assess the reliability of the individual predictions from the model, FDR for each tumor was computed by considering the cross-validation precision-recall curves (Fig. 1b). The previously known phenocopying events (*MDM2*, *MDM4* and *PPM1D* amplifications) and *TP53* deep deletions, which were held out from the training set, were largely scored as *TP53* mutated. Tumors harbouring a known phenocopying amplification were assigned higher scores than the rest of *TP53* wild-type tumors (mean score = 0.56 and 0.27 respectively, *p* = 1e-65 by t-test). Cells harbouring a *TP53* deep deletion also had higher scores (mean *TP53* deleted = 0.47, mean *TP53* not deleted = 0.27, *p* = 1e-08). We tested multiple threshold values for defining *TP53 *loss phenocopies, which expectedly yielded varying percentages of phenocopies across cancer types (Additional file 1: Fig. S1e). Our choice of threshold to detect *TP53* phenocopied tumors was set based on these known phenocopying events, conservatively, corresponding to score > 0.6, Methods; Fig. 1b).

This resulted in an empirical FDR estimated at 15% (i.e. precision of 85%), based on the known *TP53* mutations. Importantly this 15% is a conservative upper-bound estimate of FDR, since it is based on the assumption that there do not exist any unknown *TP53* phenocopying events: it classifies all high-scoring *TP53 wild-type* tumors as false positives. Conversely, using the known phenocopying events we estimate a lower-bound recall (sensitivity) of this classifier at 63% (Fig. 1b). Again, this estimate is conservatively biased, since it is not a priori known whether every copy number gain in *MDM2*/*MDM4*/*PPM1D* causes a phenocopy; some low-level gains may not have effects and thus would appear as false-negatives.

To additionally validate the classifier, we inspected the relationship between known phenocopy genes’ allele copy-number (see Methods), and the *TP53* phenocopy score. There were significant positive correlations between three known phenocopying genes copy-number, and the *TP53* phenocopy score in *TP53* wild-type tumors (Fig. 1c).

To independently validate our TP53 phenocopy classifier, we applied it to RNA-seq data from 555 tumors across 7 cancer types from the Pan-Cancer Analysis of Whole Genomes (PCAWG) study. Using the same procedure as with the TCGA dataset (FDR = 15%, recall = 80%), the FDR on this validation cohort was 8.8% (recall = 77%, Additional file 1: Fig. S1f). TP53 phenocopy scores were significantly higher for *TP53-*altered samples compared to *TP53* wild-type samples, both in a pan-cancer analysis (0.87 vs. 0.48*, p* = 2e-60, t-test) and in individual cancer types (Additional file 1: Fig. S2a,b). Similarly, known phenocopying genes scored a high phenocopy score (Additional file 1: Fig. S2c). These results were consistent with those from the TCGA dataset (0.86 versus 0.32 for TP53 altered versus *wild-type*, p≈0, t-test). Together, these data independently validate our TP53 phenocopy classifier and findings. The TP53 function loss phenocopy scores for each PCAWG tumor sample are provided in Additional file 3: Data S3.

The prevalence of phenocopying events was substantial: overall 12% of all TCGA tumor samples were redefined into a *TP53* mutated-like category (Fig. 1d) by our criteria. Different cancer types display different phenocopy frequencies (Fig. 1d), overall frequency ranging from 19% for breast cancer (BRCA cancer type) to 3% for B-cell lymphoma (DLBC cancer type); phenocopy frequencies are shown in Additional file 1: Fig. S1d. For instance, most breast cancer *TP53*-phenocopied tumors derive from previously known events i.e. the *MDM4/MDM2/PPM1D* amplifications are the most common event, while the remaining 27% of the TP53 loss phenocopies (5% of all breast cancer samples) are not associated with a known phenocopying event (proportion shown for every cancer type Fig. 1d). We note that it is still possible that individual examples of tumors may be erroneously classified as TP53-deficient at the applied score threshold. Overall, 51% of *TP53*-loss phenocopied tumor samples across all cancer types were not linked with one of the three known causal genes nor a CNA deletion in *TP53* itself, suggesting that additional *TP53* phenocopying mechanisms are commonly occurring in human tumors.

TP53 loss-of-function (LoF) mutations can result in either complete or partial LoF. Our TP53 phenocopy score distinguished between TP53 alterations that likely disrupt p53 protein activity to varying extents (Additional file 1: Fig. S2e). The VARITY and EVE variant pathogenicity scores (see Methods) predict the likelihood that a given missense variant is disease-causing. TP53 phenocopy scores were modestly but significantly correlated with these variant scores (VARITY: R = 0.16, *p* = 7e-11; EVE: R = 0.09, *p* = 1e-4), indicating that our classifier also can capture differential functional impact of TP53 missense variants. Nonsense mutations and frameshifting indels often have higher TP53 phenocopy scores than missense variants (major cancer types shown in Additional file 1: Fig. S2e).

### *USP28* deletion phenocopies a *TP53* mutated state in tumors

Prompted by the abundance of tumor samples that are functionally *TP53* null but lacking an obvious *TP53* loss or a known phenocopying event, we sought to identify other phenocopying genes across all cancer types. To this end, we designed a custom association-testing methodology that combines six different statistical tests across four different genomic data types (see Methods, Additional file 1: Fig. S3).

In brief, our methodology is based on the rationale that genes that cause a TP53 loss phenocopy via altered dosage at DNA and mRNA levels should exhibit a distinct copy number variant (“CNV” tests) and also gene expression (“GE” tests) pattern. Each of these two genomic data types is considered in two tests, one comparing *TP53* phenocopying against *TP53 wild-type* tumors, and another comparing *TP53* phenocopying against *TP53*-mutant tumors, for a total of four tests. As two additional tests, we considered external data from genetic screens across large panels of cancer cell lines [26, 27]. In particular, we tested for significant codependency scores, explaining how a knockout (“CRISPR”) or knock-down (“RNAi”) of a candidate phenocopying gene affects fitness across a panel of cell lines, when compared with the fitness profile of a *TP53* knockout/knock-down across the same panel [28, 29]. As an example supporting the use of our methodology that combines cancer genomic analysis and genetic screening data analysis, the CRISPR knockout of the known *TP53* negative regulator *MDM2* decreases cell line fitness, in a manner anticorrelated to a *TP53* knockout across cell lines. (Additional file 1: Fig. S4a).

In summary, we tested differences of tumor genomics CNV and GE patterns (two tests each as above), additionally considering “CRISPR” and “RNAi” test scores from genetic screens, for each gene, performing tests stratified by cancer type. Our final score combines the 6 tests together, providing a ranking of potential *TP53* phenocopying genes.

**Fig. 2:**
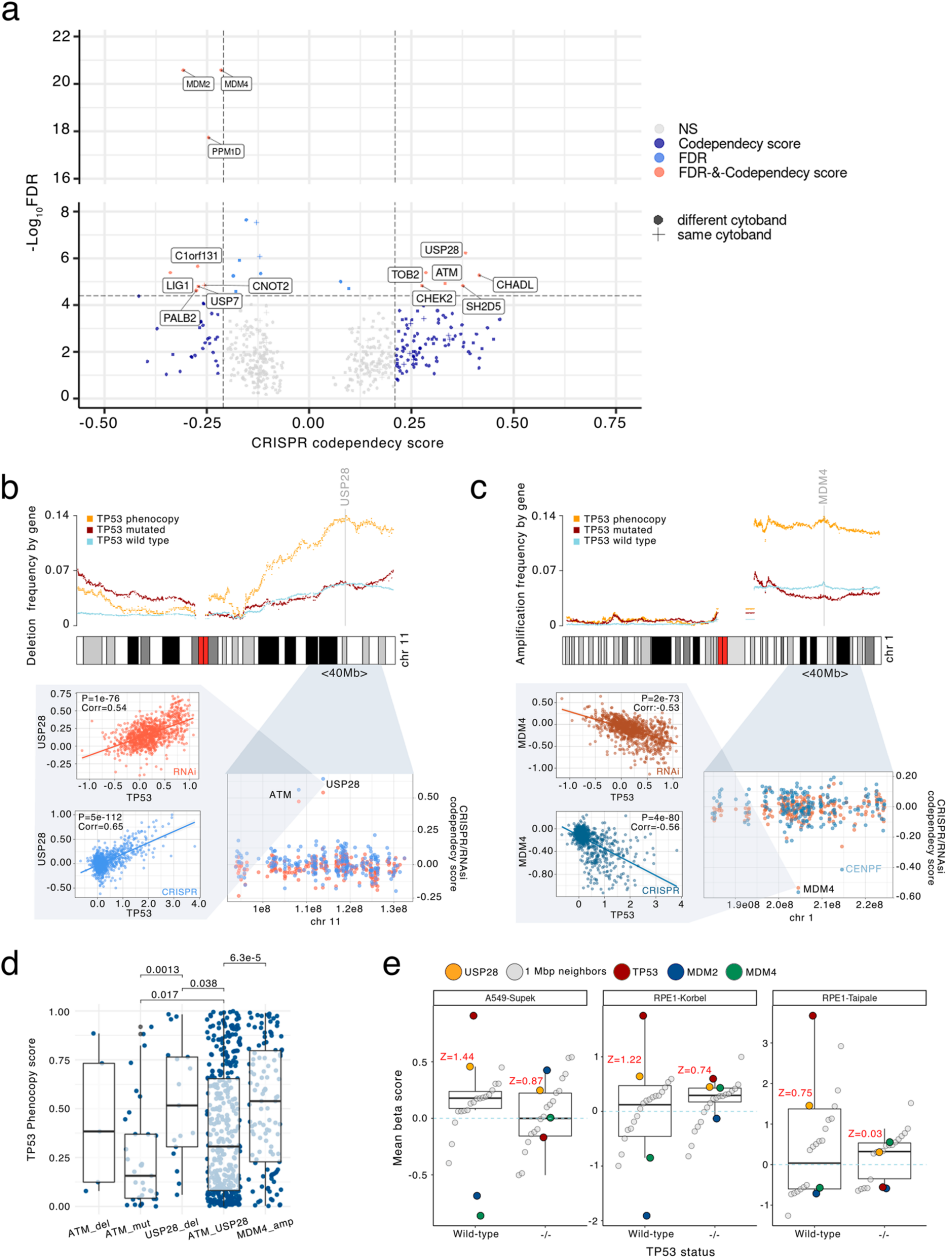
Transcriptomics scores reflecting phenocopying events can identify causal genes in CNA-affected chromosomal segments. **a** Prioritization score of genes for *TP53* loss phenocopying effects. Y axis shows gene significance (FDR) when combining six statistical tests (four cancer genomic/transcriptomic, and two based on CRISPR and RNAi screens), and further pooling *p*-values across cancer types; see Methods for details. X axis represents the effect size specifically from the CRISPR codependency test score of a gene. Crosses represent gene neighbours (same cytoband) to a known phenocopying gene. Above-threshold hits in terms of FDR and codependency score are labelled. Shown thresholds (dashed lines) for effect size and significance were determined based on scores of known phenocopy events (CRISPR score = -0.21, FDR = 4e-5, according to *MDM4* score in LUAD, see Methods). Genes with a pan-cancer CRISPR or RNAi codependecy score lower than 0.1 were filtered out. Same figure but showing the RNAi codependency score on X axis is provided in Additional file 1: Fig. S4b. **b** Top: CNV frequency in tumors, and their associations with *TP53* phenocopy transcriptomic scores, in the segment of chromosome 1 containing *MDM4*. Each dot represents one gene, while colours represent groups of tumor samples by *TP53* status. Bottom: A zoomed-in view of a commonly amplified region of the chromosome, showing the CRISPR and the RNAi *TP53*-codependency scores for each gene. The data underlying the *TP53* codependency score is shown for the top-ranking score of the region (left panels), here showing the CRISPR and RNAi fitness effects of *MDM4* disruption (Y axis) across many cell lines (dots), compared to *TP53* disruption fitness effects (X axis) across the same cell lines. **c** Same as panel **b**, but for *USP28*, a gene we identified to be associated with a *TP53* loss phenocopying via a deletion. Here, the Y axis on the top plot shows frequency of gene deletions in tumors, subdivided by *TP53* functional status, whereas panel **b** shows frequency of amplification. Bottom plots are analogous to  panel **b**. **d** Comparison of the TP53 phenocopy score of *USP28* CNV deletions (by negative GISTIC score), *ATM* deletions, *ATM* mutations and *MDM4* amplifications. Each dot represents a tumor sample. Only TP*53 wild-type* samples were considered. *P*-values by Mann–Whitney test. **e** Fitness effect of *USP28* knock-out in TP53 *wild-type* and mutant isogenic cell lines. Comparison of the mean beta score (fitness effect upon CRISPR gene disruption, y-axis) of *USP28. *, The mean beta scores of genes located within its 1Mbp immediate surroundings are shown as negative controls ("1 Mbp neighbors", see Methods). Genes *TP53*, *MDM2*, and *MDM4* are also shown as a reference. x-axis bottom labels indicate the *TP53* status of the cell line. The *USP28* Z-scores, comparing to the distribution of neighbouring genes, are plotted in red (see Methods)

As anticipated, top 3 genes by the overall prioritization score are *MDM2*, *MDM4* and *PPM1D* genes (Fig. 2a). A breakdown of our scores by different cancer types is provided in Additional file 1: Fig. S3. Following those known *TP53* phenocopying genes, the gene *USP28* was the 4th ranked gene (genes neighboring *MDM2*/*MDM4*/*PPM1D* excluded) in terms of overall statistical significance (*p* = 5.9e-07, combined across all six tests), and in particular scored highly on CRISPR codependency (pan-cancer score for *USP28* = 0.54, compared with -0.72 for *MDM2* and -0.53 for *MDM4*, breakdown by cancer type in Additional file 1: Fig. S4a). An overview of the top 20 genes according to our prioritization scores is provided in Additional file 1: Fig. S4c.We note that, in contrast to *MDM2* and *MDM4*, it is the deletions not amplifications of *USP28* that were associated with *TP53* loss phenocopying; this is reflected in the mirrored direction of the CRISPR codependency score. *USP28* encodes a deubiquitinase enzyme with substantial evidence from previous biochemistry and cell model studies that link it to p53 activity and apoptotic responses.

While it was proposed that USP28 was linked to DNA damage apoptotic responses through the ATM-CHK2-p53 pathway, recent evidence however suggests that a distinct pathway involving TP53BP1 and USP28 induces p53 and cell cycle arrest after mitotic delays [30–35]. The observation that *USP28* deletions are identified as a TP53 phenocopying event in our analysis with higher priority than *ATM* or *CHK2* alterations, suggests that loss of TP53BP1-USP28-p53 pathway drives selected events to a higher degree than loss of the ATM-CHK2-p53 pathway in many tumors. This could help tumors adapt to replication stress that can slow mitotic progression, and to tolerate supernumerary centrosomes.

Overall, diverse experimental evidence from genetic screens strongly supports our identification of *USP28* deletions as p53-loss phenocopying events, and our cancer genomic and transcriptomic analysis suggests a widespread distribution of causal *USP28* deletions across human tumors.

We also assessed how many previously unexplained TP53 phenocopying tumors (“Unknown cause” group in Fig. 1d) could be attributed to *USP28 *deletions. *USP28* deletions accounted for a subset of TP53 phenocopying tumors (12% had exclusively a USP28 deletion, 24% had a USP28 deletion and a TP53 shallow deletion) by transcriptome score that did not bear *TP53* mutations nor deep deletion and that were not associated with known phenocopy mechanisms of *MDM2*/*MDM4*/*PPM1D* amplification (Additional file 1: Fig. S4d).

Additional hits from this association study might provide promising genes for follow-up. For instance, *MSI2* was the 5th most highly prioritized gene, predicted to phenocopy *TP53* loss by amplification. *MSI2* encodes a transcriptional regulator that has been recently identified as an oncogene in hematologic and solid cancers [36–38]; we note that the *MSI2* locus is in the broader neighborhood of *PPM1D* (17q22 and 17q23.2, respectively; 3.3 Mb genomic distance). Similar results to CRISPR analyses were observed using RNAi screening codependency scores, further supporting the association of the *USP28* losses with *TP53* phenocopying, as well as the *MSI2* gains (Additional file 1: Fig. S4b). Other apoptosis-related genes such as *CHEK2* and *ATM* [39, 40] were also in the prioritized genes in our analysis, albeit at more modest statistical significance. Of note, the *TPR* gene also had a highly significant codependency score but was driven by a single cancer type (kidney) and thus with less clear relevance to diverse tumor types. Top prioritized genes and significance calls from the individual association tests are provided at Additional file 3: Data S4.

### Phenocopy scores prioritize causal genes within CNA segments

Amplifications of certain chromosomal segments (or whole arms) in case of *MDM2, MDM4* and *PPM1D* commonly underlie *TP53* phenocopies. Such CNA genetic events however often affect multiple adjacent genes, where an open question in cancer genomics is which gene or genes in the affected segment are causal [41]. We hypothesized that the known *TP53* phenocopying gene CNA segments might in some cases harbor more than one causal gene. Our genomic combination testing approach can prioritize genes with enriched gene expression and/or CNA in the *TP53* loss phenocopying group. Considered together with the prioritization by CRISPR and RNAi codependency, this method provided a plausible ranking of possible *TP53* loss phenocopying genes. Applied globally, this identified *USP28* as a novel phenocopying gene (see above). To more formally study if the *USP28*-adjacent genes could contribute to this, we considered that the same method could be applied on a local scale: by examining profiles of CNVs along chromosomes, our genomic prioritization scores would be able to single out the causal gene(s) in the segment of recurrent CNA.

As a control for this approach, we sought to confirm previously known phenocopies. Indeed, *MDM4* amplification was found to be enriched in the *TP53*-phenocopying group of tumor samples, but not in the rest of tumor groups – the *TP53* mutant and the non-phenocopying *TP53* wild-type (Fig. 2b). The local profile of this enrichment for the chromosome 1q segment 32.1 peaks at the *MDM4* gene and falls off towards its flanking genes (Fig. 2b). Our CRISPR and RNAi data analysis, consistently, indicate *MDM4* as the gene with the strongest effect in the region (Fig. 2b). As expected, similar CNA and CRISPR/RNAi profiles were observed at *PPM1D* (Additional file 1: Fig. S4e). The *MDM2* CNA enrichment score segment peak was narrower, suggesting a more focal gene amplification process (Additional file 1: Fig. S4e).

Next, we examined the local shape of the *USP28*-adjacent CNA profiles. *USP28* deletions were found to be enriched in the *TP53* phenocopying group when compared to the rest of tumor groups (2.3-fold in *TP53* w.t, 2.8-fold in *TP53* mutant). *USP28* enrichment was comparable to *MDM4* region enrichments of 2.5–3.7-fold (*TP53* wt., *TP53* mutant) (Fig. 2b,c). *TP53* phenocopying tumor samples appear to have more deletions in the *USP28* region than *TP53* wild-type (non-phenocopying) and *TP53* mutant samples. The local profile of enrichments presents a plateau-like pattern rather than a sharp peak, and *USP28* is within the top-ranked genes in the plateau, however some neighbouring genes do appear similarly so. Therefore, we further used the CRISPR and RNAi codependency scores to prioritize the causal genes in this deletion segment; these scores clearly distinguishes *USP28* from its immediate neighbours (Fig. 2c), suggesting that *USP28* is indeed the causal gene in the chromosomal segment.

Furthermore, this ‘local scan’ can be applied chromosome-wide, where we noted another small region on chromosome 11q.12.1-q1.13.1 modestly enriched with amplifications in *TP53*-phenocopying tumors (Additional file 1: Fig. S5a), thus raising our interest. However, neither the genes in this region nor other chromosome 11 regions showed a positive CRISPR codependency score of even half of the *USP28* score (Fig. 2c). Of note, the *USP28* codependency score exceeds, in absolute magnitude, the score of the known *MDM4* phenocopy (Fig. 2b,c).

In the broader neighborhood of *USP28*, the gene *ATM* seems to also be frequently deleted in the *TP53*-phenocopying tumor group, meaning *ATM* is also a candidate for the causal gene in this deletion segment at chr11 q22.3-q23.2. However, the statistical support from genomic enrichment scores (using our methodology for meta-analysis across 6 statistical tests) for *ATM* were less strong than for *USP28* (*p* = 1e-5 versus *p* = 6e-7, respectively). Consistently, comparing the RNAi and CRISPR *TP53*-codependency scores of *ATM* versus *USP28* shows a more robust effect of the *USP28* knockout (*USP28* RNAi codependency score *p* = 4.9e-112 versus *ATM p* = 3e-80, in a pan-cancer analysis; Additional file 1: Fig. S5b). To further rule out that *ATM* has the causal role in this deleted segment, we considered the cases of tumors where *ATM* is disrupted by a point mutation; unlike CNA in the *ATM* gene, these cases are not commonly genetically linked with disruptions in *USP28*. The *ATM* mutated but *USP28* wild-type tumors had considerably weaker TP53 phenocopy transcriptomic scores (median = 0.36) than the *USP28* deleted but *ATM* non-mutated tumors (median = 0.84; *p* = 0.0013 by Mann–Whitney test; Fig. 2d). The cases where both *USP28* and *ATM* were disrupted, by deletion or mutation, had very similar phenocopy scores (median = 0.73) as the *USP28* deleted but *ATM* non-mutated cases. This analysis of *ATM* mutations supports that *USP28* deletion, rather than *ATM* disruption, is the causal change in the deleted segment at chr11 q22-q23.

To validate the *USP28* finding, we analyzed an independent CRISPR data set, consisting of 3 genome-wide screens performed on *TP53* wild-type and *TP53* -/- isogenic pairs of cell lines: one on the A549 cell line pair and two on the RPE1 cell line pairs (see details in Methods). In the *TP53* wild-type background, the *TP53* k.o. increases cell fitness, as expected for a high-effect tumor suppressor gene (Fig. 2e). Thus, if the *USP28* loss was to phenocopy *TP53* loss, the *USP28* k.o. by CRISPR should also increase fitness. Indeed, compared to the 10 neighboring control genes residing within 1 Mb of *USP28*, the *USP28* k.o. has a stronger fitness effect (beta score from MAGeCK tool, see Methods) for 10 out of 10 neighboring genes in 2 out of 3 screens, and for 8 out of 10 neighboring genes in the remaining screen (Fig. 2e). For *ATM*, this effect is less pronounced (Additional file 1: Fig. S5c). In 3 out of 3 cell screening experiments, *USP28* fitness effect was stronger than *ATM* effect (1.4-fold, 2.4-fold and 2.6-fold increased beta score). To further support this finding, we asked if the fitness gain resulting from *USP28* loss is because of downstream effects on *TP53* activity. We thus considered the isogenic cells where *TP53*-/- was ablated, in which indeed the fitness gain from *USP28* k.o. was attenuated or disappeared (Fig. 2e) compared to *TP53* wild-type cells. In 2 out of 3 cell line screens, the fitness attenuation effect of TP53-/- background cells was stronger in *USP28* than in the neighboring *ATM* gene, supporting the causal role of *USP28* in that segment (Additional file 3: Data S5). Of note, in this analysis the effect sizes of *USP28* k.o. were less than the full effect of *TP53* k.o., however they were still substantial: in 2 out of 3 screens, the fitness gain of *USP28* disruption was comparable in size to the fitness loss of *MDM4* disruption (Fig. 2e).

Overall, these analyses highlight *USP28* as the likely causal gene for *TP53* loss phenocopying via deletions in the chr11 q22-q23 segment.

### Cancer type spectrum of *TP53* phenocopying events

As stated above, not every cancer type is affected by the same types of phenocopies. For instance, *MDM2* amplification phenocopy occurs often in BRCA, CESC, BLCA, LUAD and STAD but less so in HNSC, OV, MESO and  LIHC (Fig. 1d). To further elucidate the tissue-specificity of *USP28* phenocopying events, we considered the prioritization scores separately for different cancer types (Additional file 1: Fig. S3). We observed that BRCA, BLCA and LUAD were the cancer types which showed the strongest signal in our prioritization score for *USP28* phenocopies, with a suggestive signal in STAD.

To elucidate the cancer type spectrum of the *USP28* phenocopies, we considered the *USP28* amplifications as a negative control (in this gene, deletions are expected to phenocopy). In particular, we determined in which tumor types *USP28* deletions had a higher *TP53* phenocopy score than USP28 copy number-amplified samples. As expected, statistical significance (when comparing the *TP53* phenocopy score) of *USP28* copy number-neutral tumor samples versus those bearing deletions was stronger than when comparing copy number-neutral to amplifications. This supports the impact of *USP28* deletions on *TP53* loss phenocopy score. The strongest effect was found in BLCA, STAD, BRCA, LIHC and LUAD (Fig. 2e). In further support of this tissue spectrum, when CRISPR *TP53* codependency scores were checked, highest *USP28* scores were found in cancer cell lines originating from BLCA, STAD, BRCA, LIHC and LUAD (Fig. 2e). The leading codependency score was found in BLCA (effect size = 0.73, *p* = 2.2e-08) and BLCA also had the most significant value when comparing deletions to neutral copy number *TP53* phenocopy score (*p* = 4.2e-06, Additional file 1: Fig. S5d). LUAD had the second most significant codependency *p*-value (*p* = 3.8e-6), and is also highly ranked in comparison of phenocopy score between deletion *versus* neutral *USP28* CNV tumors (Additional file 1: Fig. S5d). We found a positive association between *USP28* CRISPR codependency score and the effect of *USP28* deletions in *TP53* phenocopying score across cancer types (Additional file 1: Fig. S5d). Of note, the “oncogene-tumor suppressor” dichotomy of *USP28* was reported [42], which might imply that *USP28* amplification could also result in a *TP53* phenocopy in certain contexts. However, our analysis did not support this in the majority of cancer types: out of 14 cancer types, only 3 of them had a stronger *TP53* phenocopy score in *USP28*-amplified samples than in *USP28*-deleted samples (Fig. 2e); this was the case for none of the cancer types with common *USP28* phenocopying (BLCA, STAD, LIHC, BRCA and LUAD).

Taken together, these results suggest that *USP28* deletion is a novel *TP53* phenocopying alteration that commonly affects major cancer types such as breast cancer (6.2% of total breast tumors, not counting known phenocopying events and *TP53* deletions) and also bladder, lung, liver and stomach cancer (7.6%, 7.0%, 3.8% and 2.9% cases, respectively).

### Multiple neighboring genes in CNA segment can contribute to a *TP53* deficient state

Some of the top hits found in our combined testing approach were located near to known *TP53* loss phenocopying genes such as *MDM2*. We thus hypothesized that there exist cases of ‘collaboration’ of two or more neighboring genes, affected by a single copy-number alteration event, which jointly bear upon the *TP53* loss phenotype. This would represent a special case of epistasis between two genes, caused by a single alteration that spans and affects both genes. Our data suggests that the *CNOT2* gene, residing in the *MDM2* segment in the chromosome 12q15, is a likely example of this.

**Fig. 3:**
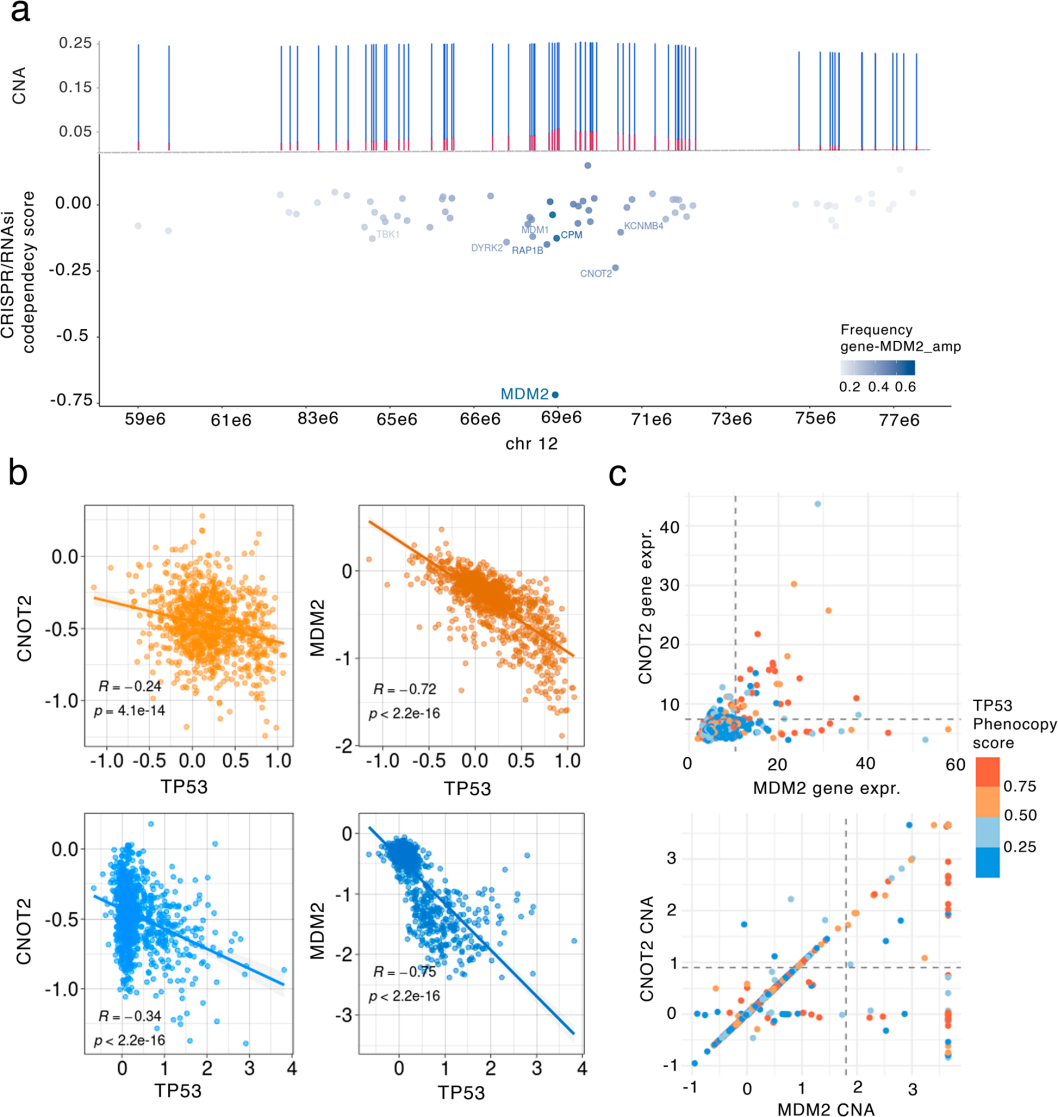
*MDM2-CNOT2* co-amplifications are associated with *TP53*-loss phenocopy score. **a** Top: Somatic CNVs of *MDM2* gene neighborhood (20 Mb segment). Y axis represents the percentage of the GISTIC CNV gain states + 1 (blue) and + 2 (red), compared to the neutral CNV state (0). Bottom: CRISPR screening *TP53*-codependency scores (y axis) shown by gene on chromosome 12 (x axis). Genes with labels have a codependency score < -0.1, suggesting possible *TP53* phenocopying effects. Color shows the frequency of CNV amplification of each gene, together with *MDM2* amplifications. **b** Co-dependency analysis underlying data from cell line panels. CRISPR and RNAi fitness effect scores for phenocopying gene *MDM2* and candidate gene *CNOT2* (y axes), and fitness effect scores for *TP53* in the genetic screens (x axes). Top plots show RNAi screening data and bottom plots CRISPR screening data. **c** Association between *MDM2* and *CNOT2* gene expression (top) and CNV status (bottom). Each dot represents a tumor sample, coloured based on the *TP53*-loss phenocopy score . Dashed lines represent the 97th quantile across genes, for each data type

In particular, in our data, *MDM2* was frequently co-amplified with *CNOT2*, in 72% of the cases of *MDM2* amplification (Additional file 1: Fig. S6a, check by cancer type at Additional file 1: Fig. S6b). Data from CRISPR and RNAi screening experiments can help resolve associations from genomic analysis, where effects of neighboring genes are in genetic linkage (here implying being jointly affected by CNA). No other gene in that neighborhood that was amplified together with *MDM2* had as high CRISPR codependency scores as *CNOT2* (effect size = -0.24, *p* = 4.1e-14, Fig. 3a,b); next best gene in the 20 Mb neighborhood is *CDK4* with effect size = -0.16, *p* = 3e-7. However, *CDK4* is co-amplified with *MDM2* in only 20% of the cases (Fig. 3a). *CNOT2*-only amplifications (i.e. without concurrent *MDM2* CNA) do not significantly associate with *TP53* phenocopy score (Pearson's *TP53* phenocopy score vs *CNOT2* CNV *p* = 0.45, effect size = -0.83, Additional file 1: Fig. S6c). More interestingly, *MDM2* CNV was not found to be associated with our *TP53* phenocopy score when *MDM2*-only amplified without *CNOT2* (Pearson´s *TP53* phenocopy score vs *MDM2* CNV *p* = 0.57, effect size = 0.09, Additional file 1: Fig. S6c). On the other hand, *MDM2-CNOT2* co-amplifications were significantly associated with a *TP53* deficiency transcriptomic score in tumors (Pearson's correlation *TP53* phenocopy score vs *MDM2* CNV *p* = 2e-05, effect size = 0.41, Additional file 1: Fig. S6c,e).

This genomic evidence is supported by recent experimental studies, indicating a role for *CNOT2* in p53-dependent apoptosis, and suggesting therapeutic potential of CNOT2 suppression (see Additional file 2: Text S1 for a summary and references). As supporting evidence, we considered fitness effects of *CNOT2* k.o. by CRISPR in various subsets of cell lines. The *MDM2*-gain but *CNOT2*-neutral genetic backgrounds had more modest fitness effects of *CNOT2* k.o. (median = -0.37) than the *CNOT2*-gain but *MDM2*-neutral genetic backgrounds (median = -0.62; *p* = 0.072 by Mann–Whitney test, Additional file 1: Fig. S6d). Consistently, the *CNOT2* k.o. by CRISPR had stronger fitness effects (median = -0.55) in the TP53 *wild-type* backgrounds than in TP53-mutant background cell lines (median = -0.45, *p* = 0.0091 by Mann–Whitney test). In other words, fitness effects of *CNOT2* disruption by CRISPR are conditional upon *MDM2* alterations and *TP53* alterations, implicating *CNOT2* in a three-way genetic interaction with the two other genes.

We hypothesized that this role of *CNOT2* in boosting the TP53-phenocopying effect of *MDM2* amplification may be variable across tissues. Our data suggests that in some cancer types *TP53* functional loss seems to rely on amplifications of both genes together, rather than solely *MDM2*, but not in all (Additional file 2: Text S2). This suggests a model where the *MDM2-CNOT2* coamplification enhances the *TP53* loss effect via a genetic interaction, and amplification of *MDM2* alone but not *CNOT2* alone able to generate a *TP53* functional loss phenotype. Gene expression profiles match this observation from CNA: having a *MDM2* and *CNOT2* co-overexpression (over the 97th percentile; *n* = 40) implies a high mean *TP53* phenocopy score (above the 84th percentile, mean phenocopy score *MDM2_CNOT2* = 0.65, Fig. 3c, Additional file 1: Fig. S6f), however less so for a *MDM2*-only overexpression (76th percentile; mean *MDM2* only score = 0.46, Fig. 3c, Additional file 1: Fig. S6f), and, expectedly, even less so for a *CNOT2-*only overexpression (73th percentile; mean *CNOT2* only score= 0.41).

This principle might extend beyond the *MDM2*-*CNOT2* pair. For instance, the *MSI2* gene, another highly prioritized hit in our combined test (Additional file 1: Fig. S6g,h,i), resides near the known phenocopying gene *PPM1D* and has the potential to boost the effects of the linked amplification of the *PPM1D* gene to cause a *TP53* deficient state. Considered jointly, these findings suggest the possibility of *TP53*-loss like phenotype being a result of multiple phenocopying events of neighboring genes resulting from a single segmental CNA.

### Detecting *TP53* loss phenocopies in cancer cell line panels

It is well known that *TP53* mutations associate with overall poorer drug response in tumors [43–45], consistent with a lower ability of *TP53* deficient cells to trigger cell cycle arrest and/or apoptosis [46–50]. We hypothesized that, in addition to conferring a generalized drug resistance, the *TP53* function loss may also modulate the association between specific drugs and alterations in their target genes. In other words, we asked whether in *TP53 wild-type* cancer cells, for instance, amplification in a particular oncogene predicts sensitivity to a particular drug, while in *TP53* mutant cells the same amplification does not associate with sensitivity. Cancer cell line drug screening panels [51, 52] provide a convenient system to test this hypothesis, because many drugs were tested systematically across both *TP53* wild-type and mutant cells originating from multiple cancer types. Considering *TP53* function loss via phenocopy score should afford additional statistical power and clarify the associations discovered; otherwise, some effectively *TP53* null cells would be erroneously considered wild-type during statistical testing, making it more difficult to identify associations.

**Fig. 4:**
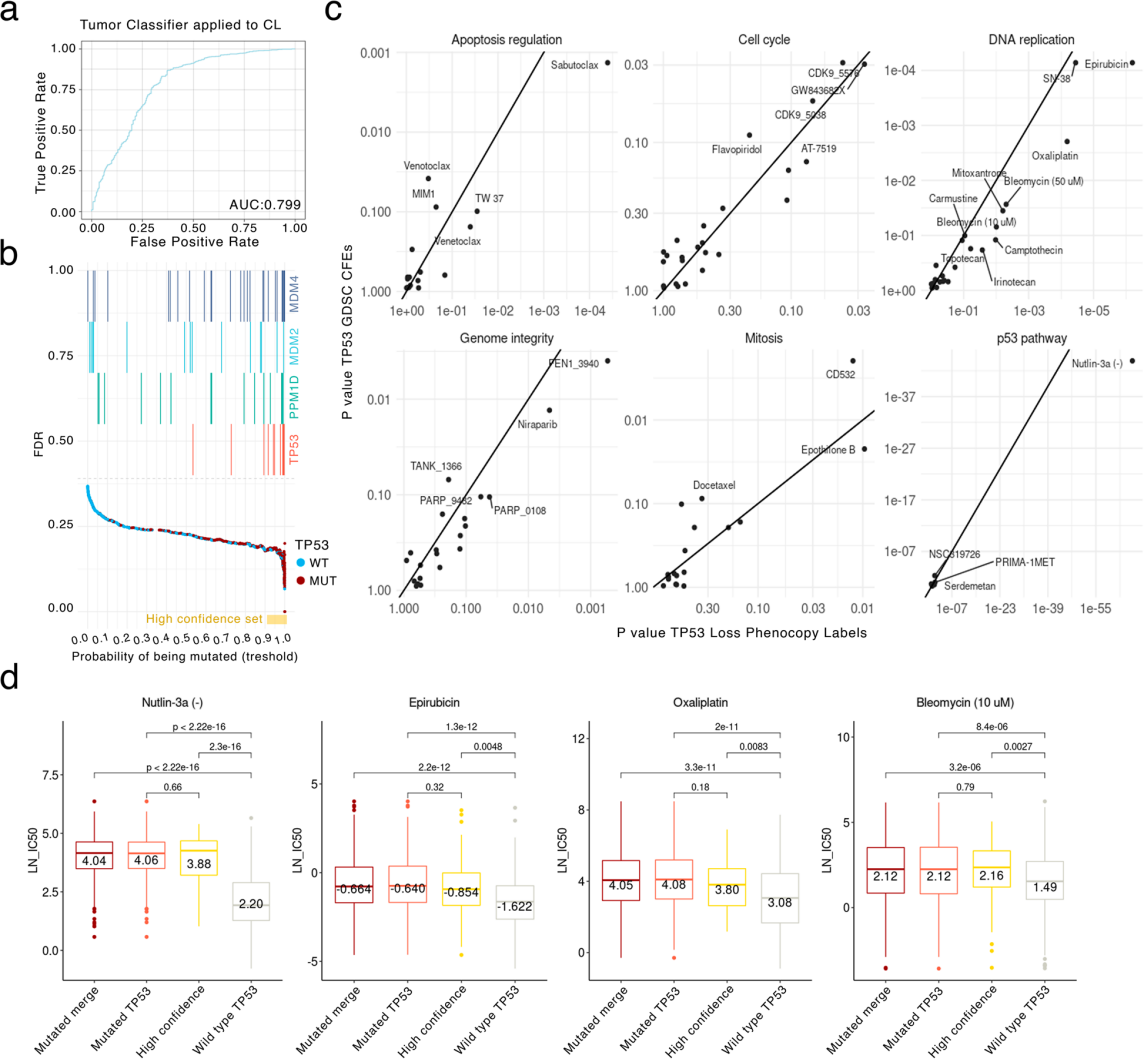
*TP53* loss phenocopy transcriptome scores predict drug sensitivity. **a** Our *TP53* functional status classifier, derived from tumor data, is applied to cancer cell line data. Receiver operating characteristic (ROC curve and its area under curve (AUC) are shown. **b** The false discovery rate (FDR) for each cell line is shown as a dot. X axis represents the phenocopy score threshold at which each cell line would be classified as *TP53* functionally deficient. Yellow horizontal bar represents the range for our definition of a high-confidence set of TP53 phenocopying cell lines (FDR = 0.18, threshold = 0.93). In the top part of the plot, cell lines harboring deletions of *TP53*, and amplifications of known phenocopying genes *MDM4*, *MDM2* and *PPM1D* are marked. **c** *TP53* status—drug sensitivity associations. Each panel represents the drugs targeting genes within a given pathway. Each dot represents an association of a drug with two possible *TP53* functional status labels: X axis with the *TP53* phenocopy score and Y axis with the *TP53* mutational status (“CFE” labels by the GDSC, see Methods). *P*-values are from a pan-cancer regression test for association between a given drug log IC50 with the *TP53* status. Associations with FDR < 0.25 are labeled. **d** Distributions of log IC50 values for several example drugs where *TP53* status is known to confer resistance. The X axis illustrates the different categories based on *TP53* mutated status (“Mutated *TP53*”), wild type *TP53* (“Wild-type TP53”) and a high *TP53* phenocopy score (“High confidence”); the “Mutated merge” is a combination of the two. P-values from statistical tests results comparing the groups (Mann–Whitney test, two-tailed) are plotted on top. Median values are provided inside each box

First, we aimed to generalize our tumor *TP53* phenotype classifier to cancer cell lines. Because cell lines exhibit strong global (i.e. affecting many genes) shifts in gene expression patterns, compared to their tumor tissue of origin, we applied an adjustment methodology as in our recent work [53] using the COMBAT tool [54]. Upon adjusting gene expression from cell lines in the CCLE and GDSC panels to make it comparable with TCGA tumors (see Methods), we applied the *TP53* classifier and obtained ranked scores. As a positive control, the classifier assigned a significantly higher *TP53* phenocopy score to *TP53* mutated cell lines (mean *TP53*_wt = 0.43, *TP53*_mut = 0.83, *p* = 1.1e-49 by t-test), therefore cell line data served as an independent validation set for the classifier. Of the 610 cell lines labeled as *TP53* mutant based on genomic sequence (see Methods), 87% were classified as having a *TP53*-loss phenotype (Fig. 4a), suggesting a reasonable ability of the classifier trained on TCGA tumor transcriptomes to generalize to cell line data.

Similarly as in tumors, a notable fraction of cell lines were apparent false positives, labelled as *TP53* wild-type by the genomes, but classified as *TP53* deficient using the phenocopy score. We subdivided these apparent false positives into a high-confidence phenocopying set and the rest; the *TP53* phenocopy score of the *TP53* deep-deleted tumor samples was used as the threshold (see Methods). The high-confidence set was composed of 76 cell lines (FDR = 18%, see Methods, Fig. 4 B). Only 79% of the cell lines labelled as *TP53* wild-type genetically were also classified as *TP53* wild-type by the phenocopy score, suggesting that *TP53*-loss phenocopying events are common among cancer cell lines. For comparison, this percentage was 77% in cancer samples.

Some of the apparent false positive cell lines had a *MDM2*, *MDM4* or *PPM1D* amplification or a *USP28* deletion (43 out of 109, 39% of the high-confidence set). Cell lines harboring one of these CNA in known phenocopying genes had higher scores than the rest of *TP53* wild-type cell lines (mean score = 0.58 and 0.37, respectively; t-test *p* = 5.4e-5, Additional file 1: Fig. S7a). Cells harboring a *TP53* deep deletion (90th percentile of CNA scores) also had higher phenocopy scores than samples without deletion (mean score = 0.78 and 0.33, respectively, t-test *p* = 5.4e-8). 28% of the cell lines in the high-confidence harbor a *TP53* deep deletion (22 out of 76, 90th percentile of *TP53* deletion CNA). These data support that the apparent false positives are often *bona fide TP53* phenocopying events in cancer cell lines. All *TP53* phenocopy scores and cell line functional *TP53* status information are provided in Additional file 3: Data S6.

### Effects of *TP53* on drug resistance are clarified by *TP53* phenocopy scores

Next, we considered the GDSC drug response distributions for various drugs, in light of the *TP53* functional status as determined by the *TP53* mutations, and additionally as determined by our TP53 phenocopy scores. To identify drugs to which response is affected by *TP53* functional status, we predicted drug response (log IC50) values of 449 GDSC drugs individually, using *TP53* status as an independent variable (see Methods).

For most of the tested drugs (105 out of 188 drugs that were significantly associated at < 25% FDR, pan-cancer), the associations with *TP53* had a lower FDR when testing using *TP53* phenocopy score, over when using the *TP53* CFE labels (denoting *TP53* mutations which alter gene function) (Fig. 4c, effect size at Additional file 1: Fig. S7b). For the drugs that affected pathways related to *TP53*, this effect of improved significance by using the phenocopy score was prominent (hits with FDR by *TP53* phenocopy score < FDR by *TP53* CFE labels: DNA replication, 12/12 drugs; genome integrity, 8/10; p53 pathway, 3/5; apoptosis regulation, 4/6; cell cycle, 4/7; Additional file 1: Fig. S7c). As a negative control, randomized *TP53* labels were not significantly associated with any drug. As a positive control, the drugs known to be affected by *TP53* status such as nutlin-3a (effect size = 1.48 vs 1.01, *p* = 6.7e-68 vs 1.2e-44) or bleomycin (effect size = 0.25 vs 0.16, *p* = 0.009 vs 0.07), exhibited a stronger association with the TP53 score than with *TP53* CFE mutation labels (Fig. 4c).

Next, we examined the IC50 drug sensitivity values towards all drugs together, considering the different groups of cell lines defined by our TP53 functional status classifier (Additional file 1: Fig. S7d). Here, the mean IC50 values of high-confidence *TP53* phenocopying cell lines is more similar to the *TP53* mutated cell-lines than to the *TP53* wild-type cell lines. In drugs known to be affected by *TP53* status, such as bleomycin (Fig. 4d), IC50 values were not notably different between *TP53* mutant and the *TP53* phenocopying high-confidence cell lines. All drug associations effect size and *p*-value are plotted in Additional file 1: Fig. S8a,b. Cancer type-specific associations are shown at Additional file 1: Fig. S9.

Taken together, the above analyses support the utility of the phenocopy score in identifying *TP53*-associated drug sensitivity or resistance, and also support that our tumor-derived classifier is able to generalize to cancer cell line transcriptomes to detect a phenotype of functional *TP53* loss.

### Associations between drug sensitivity and genetic markers are modified by *TP53* status

**Fig. 5:**
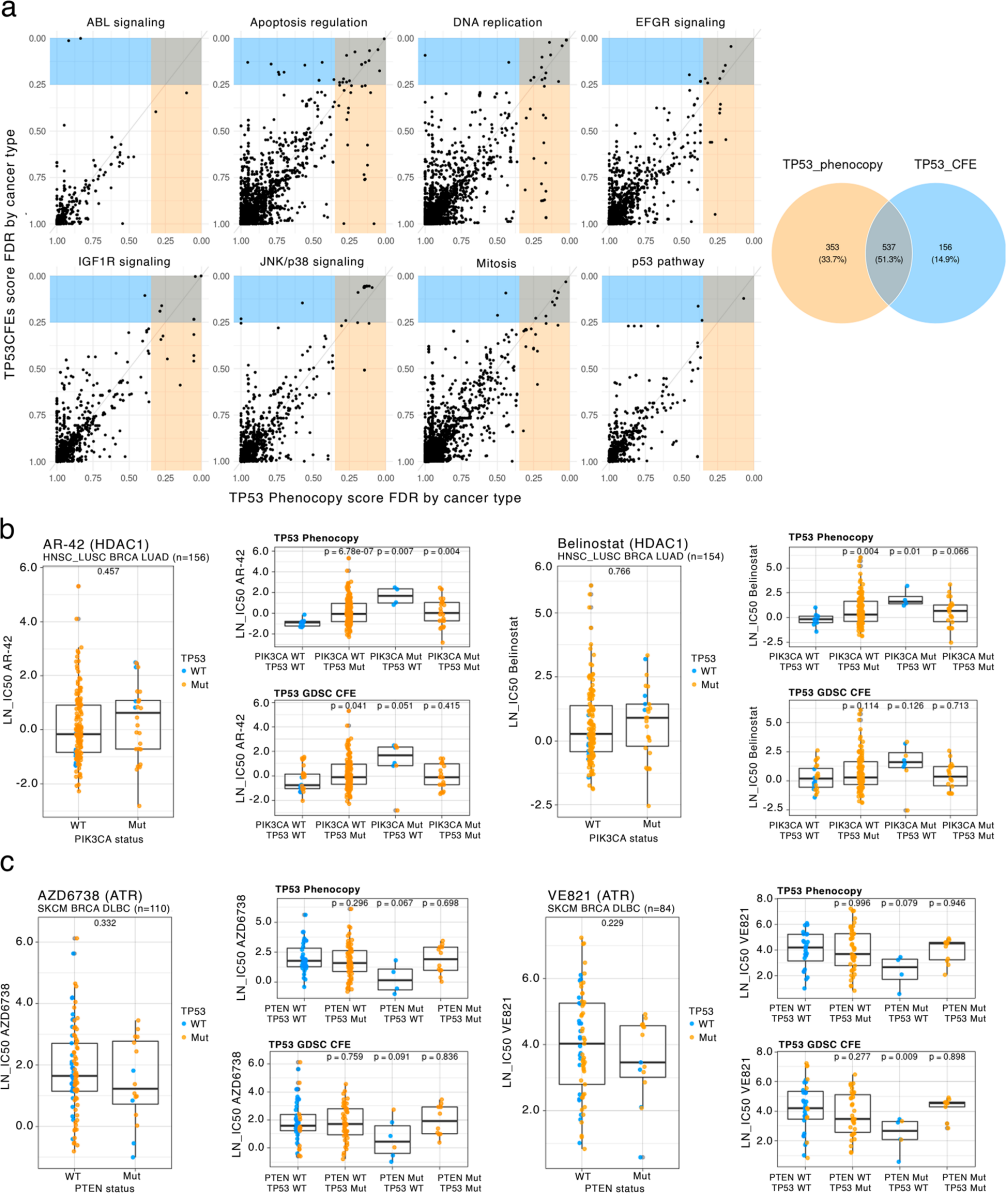
Associations between drug response and genetic markers are commonly modified by *TP53* functional status. **a** Identifying associations of mutations in various genes with antitumour drug sensitivity, controlling for *TP53* status. Each panel represents a pathway targeted by drugs, and each dot represents a gene * drug *  cancer type combination. Associations are conditioned on *TP53* status by including an interaction term in the regression, where the Y axis shows associations using *TP53* mutated status using GDSC labels (*TP53* CFEs), while the X axis represents the same using *TP53* loss phenocopy score-based labels. Yellow-shaded area contains associations with FDR < 0.25 for TP53 phenocopy labels, and blue-shaded area shows the same for *TP53* CFE labels. Total counts of significant associations in shaded areas are shown in the Venn diagram. **b** Association of *PIK3CA* mutation status with HDAC1-targeting drugs (AR-42 and CAY10603), after controlling for *TP53* status. Large plots show the association without stratification by *TP53* labels. “CFE” denotes mutated (Mut) or wild-type (WT) *PIK3CA* state. An association *p*-value above each box is by Mann–Whitney test. Each dot is a tumor sample belonging to one of the cancer types listed above the panel. Dots are colored according to *TP53* phenocopy score labels. Small panels represent the same association but upon stratification by TP53 status; top row, stratification using TP53 phenocopy score labels; bottom row, using *TP53* GDSC CFEs (“cancer functional events”, impactful mutation status, see Methods). The X axis represents tumor samples stratified by both the *PIK3CA* and *TP53* status. PIK3CA status groups refer to *PIK3CA* stratification (WT, Mut) ignoring TP53 status. Labels should be interpreted as follows: “PIK3CA(WT/Mut) * TP53 (WT/Mut)” refers to stratification by *PIK3CA* status (CFE i.e. driver mutation status), using TP53 phenocopy labels (top) or *TP53* CFEs (bottom). **c** Association of *PTEN* mutation status with ATR inhibitor drugs (AZD6738 and VE821), after controlling for TP53 status. Organization of the plots matches panel **b**.

A central goal in personalized cancer medicine is to discover actionable mutations, which are used as genetic markers to decide which therapy to apply. Based on the role of *TP53* mutations in dysregulating various processes relevant to tumorigenesis, we hypothesized that various druggable cancer vulnerabilities may be conditional upon *TP53* functional status. To investigate, a regression was fit to predict activity (log IC50) for each drug, from cancer type and each cancer gene mutation status (via the CFE classification, see Methods), and additionally introducing *TP53* status (either via *TP53* mutation (“CFE”), or via TP53 phenocopy score) as an interaction term in the regression. The TP53 phenocopy score was binarized (> 0.6 used as threshold) to be able to compare fairly with the TP53 mutation status. Comparing TP53 phenocopy association FDRs against *TP53* mutation association FDRs suggested that the application of phenocopy score allowed to more confidently identify the drug-gene associations where *TP53* status modulates the effect size (see the comparison of FDR values in Fig. 5a, broken down by pathway that targets the drug). Out of the identified three-way associations (gene * drug * *TP53* status), 34% were identified only by using the *TP53* phenocopy score, but not by the *TP53* mutation status (Fig. 5a). For comparison, only 15% three-way associations were uniquely identified by *TP53* mutation status but not by phenocopy score. We provide a tally of all gene-drug associations that were conditional upon *TP53* in Additional file 1: Fig. S10a, and a per-gene comparison of associations identified with *TP53* phenocopy score labels, versus those identified by *TP53* mutational status, in Additional file 1: Fig. S10b.

Next, we aimed to select the more robust associations. To this end, we applied the “two-way” testing approach to identify replicated drug-marker links [55] seen  across two or more drugs that share the same target protein or pathway. These were tested separately for individual cancer types, comparing *TP53*-deficient versus wild-type cell lines. Here, this “two-way” randomization test [55] was further modified to be able to detect interactions with a third factor, the *TP53* functional status. As an additional criterion ensuring confidence of associations, only the hits that recur in more than one cancer type were taken into consideration (as a trade-off, this will cause tissue-specific associations to be missed). Stratifying by *TP53* functional status, we identified a number of drug-gene CFE associations that would not be significant if ignoring the *TP53* status during testing (60% of total, < 25% FDR, Additional file 1: Fig. S10c). This corresponds to a total of 2303 associations of a drug to specific gene mutational status by cancer type (total number of tests ignoring *TP53 n* = 486,417 versus *n* = 402,945 controlling for *TP53* status, Additional file 1: Fig. S10c,d). Of these, 133 associations were found in both approaches, but had a lower FDR when considering *TP53* stratification (mean FDR = 15% versus 19% if not stratifying, *p* = 5e-08); all associations from the “two-way” replication test are listed in Additional file 4: Data S7.

### Sensitizing effects of driver mutations to HDAC and ATR inhibitors are modulated by *TP53*

Studies suggested a role of the drug AR-42 (a HDAC1 inhibitor) in prolonging p53 life and triggering apoptosis [56, 57]. We hypothesized that, if p53 activity is impaired, this effect of HDAC inhibitors may be altered. Interestingly, our testing reveals that mutations in the *PIK3CA* oncogene are associated with sensitivity to HDAC1 inhibition in a manner conditional upon *TP53* mutation. In other words, when *TP53* is functional, the resistance to HDAC1 inhibitor AR-42 due to *PIK3CA* mutation is higher than when *TP53* is mutant or otherwise inactivated as indicated by phenocopy score (*TP53* wild-type *A PIK3CA_mut regression coefficient test *p* = 0.005, Cohen’s d = 1.3, *TP53* mutant PIK3CA regression coefficient test *p* = 0.08, Cohen d = -0.38, Fig. 5b). We would not identify this association with AR-42 while ignoring *TP53* status (test on regression coefficient only using *PIK3CA* mutation status *p* = 0.67, Cohen d = -0.08). In particular, in LUAD the difference in AR-42 sensitivity (median of normalized log IC50 across cell lines) between *PIK3CA* mutant and *PIK3CA* *wild-type* is hardly evident: 0.26 versus 0.24 respectively, while in *TP53*-functional LUAD this difference is -0.43 (*PIK3CA* wild-type) versus 0.35 (*PIK3CA* wild-mutant). This response is observed across three different HDAC inhibitors and in three different cancer types. For instance, AR-42 and belinostat were found significantly associated with *PIK3CA* mutation in HNSC + LUSC (here considered jointly because of known molecular similarities of the cancer types), in BRCA, and in LUAD cancer types (Fig. 5b). Similarly, the AR-42 association with *PIK3CA* mutation was supported in the HDAC1-targeting drug CAY10603 (Additional file 1: Fig. S11b). Furthermore, in an independent drug screening dataset, PRISM [51], we were able to recover these associations (Additional file 1: Fig. S11b). This example illustrates how being aware of *TP53* functional inactivation status allows to detect drug-gene associations that may be specific to the *TP53 wild-type* or to the *TP53* deficient backgrounds but not both.

We also noted that the HDAC1i resistance/PIK3CA mutation association (conditional upon *TP53* functional status) was only recovered when controlling for *TP53* phenocopy score, but not when using simply the *TP53* mutation status (per CFE method, see Methods) as an interaction term (belinostat IC50-PIK3CA mutation Mann–Whitney test, in the *TP53* mutation wild-type background *p* = 0.13, while in *TP53* w.t. phenocopy labels background *p* = 0.01, Fig. 5b). This example illustrates how the use of *TP53* phenocopy scores provides additional power to identify drug-gene associations, as already indicated by the comparison of FDR scores for many associations above (Fig. 5a).

Recent reports have pointed out the potential therapeutic benefit of ATR inhibitors such as VE-821 or VE-822 in PTEN-deficient breast, glioma and melanoma cells [58, 59]. ATR is a crucial kinase regulating DNA repair and safeguarding genome integrity. ATR inhibition in PTEN-deficient cells was associated with accumulation of DSBs, cell cycle arrest and induction of apoptosis [58, 59], thus based on these phenotypes we hypothesized that the TP53 functional status may modulate this effect. Inspecting our data supports that the ATR inhibitors VE-821, VE-822, and AZD6738 were associated with a lower fitness in *PTEN*-mutant cells of the SKCM, OV, BRCA and DLBC cancer types (Fig. 5c, Additional file 1: Fig. S11d). This effect was however revealed only when *TP53* status was taken into consideration, since p53-deficient cells had an increased survival that obscured this association of PTEN and ATRi (Fig. 5c, Additional file 1: Fig. S11d). While significance of the *TP53* interaction term was not reached in this particular example, probably because the number of cell lines with a *PTEN* mutation (but *TP53* wild-type) was low, nonetheless the association of ATRi IC50 values was found to be more robustly significant in a *TP53* wild-type context than in a *TP53* deficient context. This means there was a more prominent difference in cell fitness upon ATRi treatment comparing *PTEN*-mutated to *PTEN* wild-type cells in a *TP53*-proficient background (*TP53* wild-type IC50-PTEN Cohen’s d = -0.41 vs *TP53* deficient AZD6738 IC50-PTEN Cohen's d = -0.05).

Overall, above we highlighted two examples where *TP53* functional status modulates the association between HDAC1 inhibitors and *PIK3CA* driver mutations, and ATR inhibitors and *PTEN* driver mutations. There were however many other significant associations involving *TP53* status, cancer driver gene mutations (or CNA) and activity of drugs (listed in Additional file 4: Data S7), for example the association between *PIK3R1* mutations and sensitivity to MET inhibitors (Additional file 1: Fig. S11c).

To estimate the importance in considering TP53 status in discovering drug associations more generally, we considered overlap in associations recovered when *TP53* status was accounted for *versus* when *TP53* status was ignored. Only 14% of significant associations of a given molecular target to driver gene alteration status were shared between two analyses (Additional file 1: Fig. S10c), indicating that considering *TP53* status strongly alters the drug-gene links that can be recovered from statistical testing of drug screens on cancer cell lines. The TP53 status-aware testing recovered a higher number of associations (*n* = 12,150 versus 7853, both at < 25% FDR). We also noted this effect depended on the particular gene: drug responses in genes such as *KRAS* or *TP53BP1* are well explained by gene mutational status alone, not benefitting from *TP53* stratification (Additional file 1: Fig. S11a). Nevertheless, for most genes, their drug associations were more confidently retrieved when *TP53* status was accounted for (e.g. *BRAF*, *HRAS*, *ATM*, *APC*; *n* = 18 genes total). Overall, the above data suggests that *TP53* functionality should be considered when matching drugs to cancer patients based on the various driver gene mutations in their tumor, and that this *TP53* functional status should preferentially be estimated via a phenocopying score rather than *TP53* gene mutations.

## Discussion

Disabling the master tumor suppressor gene *TP53* provides cancer cells with important advantages such as avoiding cell cycle arrest or apoptosis upon replication stress or DNA damage. Because TP53 acts as a transcription factor controlling expression of hundreds of genes, a functional read-out of *TP53* activity can be obtained using gene expression data, both at the level of RNA or at the protein level [20–23]. These gene expression-based scores of TP53 function have potential clinical relevance in predicting cancer aggressiveness/patient survival and therapy response [22, 23, 60, 61]. In this study, we developed a robust global transcriptome score of *TP53* deficiencies, and applied it to ~ 7,000 tumors and ~ 1,000 cancer cell lines, to answer three questions.

Firstly, we asked how common are the *TP53*-mutation phenocopying events across human cancers. We estimated a 12% frequency of *TP53* loss phenocopies, compared to a 58% prevalence of *TP53* mutant tumors. In some cancer types such as BRCA and BLCA, the *TP53* phenocopies may constitute a high fraction of 19% and 16% tumor samples, respectively, suggesting that the *TP53* status of tumors should preferentially be measured via a functional readout (here, transcriptome-wide signature) rather than considering only the *TP53* gene mutations. Supporting this notion, a recent study using a four-gene expression signature of *TP53* activity demonstrated that this significantly predicts patient survival across 11 cancer types, and that in the majority of those it performs better than considering *TP53* mutations [22].

Secondly, given the high prevalence of *TP53* phenocopies, we asked if there exist additional genetic events that are associated with these phenocopies. We developed a method that considers CNA profiles jointly with gene expression in tumors, further integrating experimental data from CRISPR and RNAi screens, which confidently identified the *USP28* gene deletion as a common *TP53*-loss phenocopying event. This is relevant for at least five cancer types: BLCA, STAD, BRCA, LIHC and LUAD, and affects 2.9%-7.6% tumor samples therein. The same statistical methodology also highlighted additional genes neighbouring the known phenocopies *MDM2* and *PPM1D* – the *CNOT2* and *MSI2* genes, respectively – which are often co-amplified with the ‘primary’ gene in the CNA gain segment and may boost the resulting *TP53*-loss phenotype. Our analysis provides an example illustrating a more general principle of how molecular phenotypes (here, a transcriptional signature and fitness effects from a CRISPR screen) can be used to identify causal genes in a CNA segment. Our consideration of CRISPR k.o. screening data allowed us to circumvent the confounding effects of genetic linkage within the recurrent, phenocopying CNA segments, because the CRISPR reagent effects are not strongly coupled to the localization of the genes. Related genomics methodologies could be applied in future work to interrogate various recurrent CNA events observed in tumors, for which the causal gene(s) are often not known with confidence.

Thirdly, we asked if a better measurement of the *TP53* functional inactivation status may be helpful for predicting cancer cell response to antitumor drugs based on genetic markers. Given that *TP53* deficiencies have myriad downstream consequences on the cell, including e.g. suppression of cell cycle checkpoints, or inactivation of various DNA repair pathways [4] it is conceivable that the *TP53* background may affect the activity of various drugs to kill cancer cells, including drugs targeted towards a particular driver mutation outside of *TP53* itself. We searched large data sets for three-way statistical interactions involving *TP53* status, each drug activity, and each mutated cancer driver gene. This suggested for instance that *TP53* status modulates the selective resistance towards HDAC1-inhibitor drugs in *PIK3CA*-mutant cells. The associations we identified were filtered to retain those supported in multiple compounds targeting the same protein or pathway; enforcing agreement across multiple measurements may allay concerns of reproducibility in cell line screening databases [62–64]. Recent work by us and others [55, 65] has used statistical methods to integrate over diverse screening datasets, considering drug and CRISPR genetic screens jointly, thus improving reliability of drug-target association discovery. Our robustly supported set of drug-target gene links that may be modulated by *TP53* status (Additional file 4: Data S7) provides a comprehensive resource for follow-up validation of the *TP53* functional status in modulating gene-drug associations.

The statistical model that we employed to identify *TP53* loss phenocopying events draws on the expression levels of 217 genes, and is largely portable across various human tissues. Given that the model’s predictive accuracy is high (demonstrated using cross-validation and using an independent data set of cancer cell line transcriptomes), the errors it makes may be of interest. While the apparent false-positives are often *TP53* loss phenocopies using still-unknown mechanisms, extending the *USP28* example addressed in this study, it would also be interesting to look into the apparent false negatives in future work. These *TP53*-mutant tumors classified as *TP53* *wild-type*-like by our transcriptome score were not considered here, because of their relatively modest number, making statistical analyses difficult. Going forward, analyses of genomes from larger cohorts of cancer patients may provide enough examples to reveal mechanisms of re-establishing activity of mutated *TP53* in certain cancers. Conceivably, this may happen by normalizing expression of the *TP53*-downstream genes which have been dysregulated by the *TP53* mutation; understanding these events may inspire new avenues for therapy of TP53 mutant tumors.

The general statistical approach presented here could be applied beyond *TP53* also to other driver gene phenocopying events which may occur in tumors. For instance, RAS pathway activation transcriptomic scores were proposed [21], and similarly so the homologous recombination DNA repair scores were proposed based on mutational signatures [66, 67]. Conceivably, other cancer pathways may be similarly addressed as well, systematically analyzing their distribution across tumors to identify phenocopying events, as well as their implications to drug response prediction, as we have done here for *TP53* phenocopies.

## Conclusions

Our study provides insights into the prevalence and implications of TP53 deficiency in human cancers, which often happens by phenocopying, thus highlighting the need to consult a functional readout of TP53 activity in precision medicine efforts. Integration of a transcriptomic signature of TP53 inactivity with experimental data from CRISPR and RNAi screens identified *USP28* gene deletions as a common TP53-loss phenocopying event in tumors, and in addition we identified auxiliary genes neighbouring *MDM2* and *PPM1D* that may enhance their phenocopying effect. Furthermore, our study suggests that a measurement of TP53 functional status can improve the prediction of cancer response to antitumor drugs, such as HDAC and ATR inhibitors.

## Methods

### Data collection and preparation

#### Gene expression and Copy Number Alteration (CNA) data

We downloaded gene expression data (transcripts per million, TPM) from GDC Data Portal [68] for human tumor samples (TCGA) and from GDSC [51] and CCLE [69] for cell line samples (CL).

We filtered out genes with missing values in more than 100 samples and selected the overlapping genes between cell lines and tumors. Cancer types with less than 10 samples were filtered out. Low expressed genes were removed (TPM < 1 in 90% of the samples) and applied a square-root transformation to TPM. Cancer types. Tumors with less than 10 samples were filtered out. In total, we have 12,419 features for 966 CL samples and 9149 TCGA samples.

Tumor purity estimates were obtained from TCGA ABSOLUTE (using Tumor.purity function, R package TCGAbiolinks 2.25.3). Samples with top quality (ABSOLUTE score = 86) were removed for correlation testing.

We collected CNA from GDC Data Portal [68] for TCGA samples and from DepMap [63] for CL samples.

For external validation, the RNA-Seq data and CNV data for PCAWG [70] was downloaded using Xenahub [71]. Samples shared between TCGA and PCAWG datasets were removed from PCAWG. Only the genes present in the TCGA dataset were kept in PCAWG RNAseq data. RNAseq data was square root transformed. Cancer types that were considered equivalent between the two datasets were the following: ESAD/ESCA, CLLE/DLBC, MALY/DLBC, RECA/KIRC, PACA/PAAD, LIRI/LIHC. In total, we used 12,419 gene expression features for 555 PCAWG tumors across 7 cancer types.

#### Data alignment between tumors and cell lines

In order to later generalize the model to cell lines we proceed to align TCGA and CL data. For this, we applied ComBat, a batch adjustment method, to account for intrinsic differences between tumor signal and cell lines signal [54]. For the alignment of TCGA and CL data, we first applied quantile normalization (normalize.quantiles function, preprocessCore R 1.48.0 package) using tumor data as reference and then applied ComBat (ComBat function, R package sva 3.32.1). Each group (TCGA, GDSC or CLLC) was treated as a different batch. We proceed similarly using PCAWG gene expression data.

#### *TP53* status label (according to GDSC)

TCGA Pan-Cancer Atlas somatic mutation data were extracted from the MC3 Public MAF (v0.2.8) data set [72]. We followed the Iorio et al. methodology [73] to determine bona fide *TP53* mutations (0:wild type, 1: mutated). We identify recurrent variants that are likely to contribute to carcinogenesis. We considered mutated variants: non-synonymous missense mutations, indels (in frame insertions and deletions and out of frame insertions and deletions), nonsense mutations and specific splice-site mutations (such as “p.X125_splice”). Samples without any of these mutations annotated were considered *TP53* wild type. In only 5% of the cases (179 out of 3416) our labels differed from the ones provided by Iorio et al. In total, we obtained *TP53* labels for 7788 TCGA tumors. TP53 variant pathogenic scores were obtained from the EVE data portal [74] and from VARITY data portal [75]. For the validation dataset, TP53 mutations were downloaded from PCAWG XenaHub (*simple somatic mutation (SNVs and indels)—coding driver mutations*, [70]).

#### *TP53* score classifiers in human tumors

We used the aligned human tumor data to train a supervised elastic [20–23] net penalized logistic regression (using cv.glmnet function with alpha = 0.5, R package glmnet 4.0–2) classifier with cyclical coordinate descendent optimization [76]. The choice of Elastic net penalization aims to deal with two concerns: the large number of variables can lead to high complexity (overfitting) and the feature multicollinearity. Elastic net regressions are seen as a good trade-off that benefit from the dimensionality reduction provided by Lasso penalization while keeping as many informative variables as possible (Ridge penalization). Of note, these three regularization methods yielded similar cross-validation accuracy: Elastic net (i.e. alpha = 0.5) AUC 0.960, Lasso (i.e. alpha = 1) AUC 0.965, and Ridge (i.e. alpha = 0) AUC 0.952, suggesting that the default alpha = 0.5 in Elastic net method is a reasonable choice. The model is trained using RNAseq data (X matrix) to infer *TP53* status (Y matrix). As a reference (Y) during training we used *TP53* mutation status labels.

For the training set, we excluded the tumor samples that have an amplification (not neutral, > 0, according to GISTIC CNA thresholded calls downloaded using FirebrowseR package, Analyses.CopyNumber.Genes.Thresholded function) in previously known *TP53* phenocopying genes (*MDM2*, *MDM4*, *PPM1D*) or a deep deletion of *TP53 (n* = *2065)*, to prevent the model from relying too much on dosage effects of these genes, instead of the downstream response. Known phenocopies and TP53 gene expression were removed from the training dataset with the same purpose.

In addition, to control for cancer type specific signals we included cancer type as a dummy variable. To control for class imbalance, we included weights in the classifier.

The model learns a vector of gene-specific weights that better classifies *TP53* status. The score from the models determines the probability of a given tumor of being *TP53* deficient. Optimization of the penalized regression formula and further details of the classifier can be consulted at [76].

#### Leave-one-cancer type-out (LOCTO) classifier

We used PCAWG gene expression data aligned with TCGA data using ComBat to train an Elastic Net classifier with the same features and structure as above. The model is trained using RNAseq data (X matrix) to infer TP53 status (Y matrix). In this case the models were fit excluding from the training data all examples from one cancer type, iterating over cancer types. Metrics and curves from Additional file 1: Fig. S1a were calculated using the held out cancer type data.

#### Assessment of the classifier and calculation of FDR score

Using 90% of the training set and 5 balanced folds (balanced based on *TP53* mutational state) we performed cross-validation. We measured the performance of the training set (folds used for training) and the testing set (10% held out). Areas under the Receiving Operating Curve (AUROC) and the Precision Recall curve (AUPRC) were calculated for both training (cross-validation) and testing sets.

FDR was calculated by sample using each sample probability score from the classifier as threshold for determining positive and negative samples FDR = false positive / (false positive + true positive). Samples harboring an amplification (GISTIC thresholded amplifications, FirebrowseR package, Analyses.CopyNumber.Genes.Thresholded function) of known phenocopying genes (*MDM2*, *MDM4*, *PPM1D*) or *TP53* deletions (GISTIC thresholded deep deletions, FirebrowseR package, Analyses.CopyNumber.Genes.Thresholded function)) were considered as true positives when calculating FDR.

In Fig. 1b, density of known phenocopies was calculated using *MDM4*, *MDM2*, *PPM1D* (amplifications) and *TP53* (deletions) CNA over/under the 95/0.05 th quantile. All *TP53* Phenocopy scores (probabilities of being *TP53* dysfunctional) are provided at Data S2.

For the validation data (PCAWG), the same procedure was followed. In this case, we used the downloaded consensus CNV data to determine samples that were amplified for MDM4/MDM2/PPM1D or deep-deleted for TP53.

Precision and recall in the TCGA and PCAWG dataset were obtained similarly to FDR. MDM2/PPM1D/MDM4 amplification and TP53 deep deletion were considered to be positive cases.

The classifier coefficients were analyzed using the GO enrichment tool ShinyGO [77]. The 12,419 genes from the gene expression matrix with a coefficient equal to zero were used as background. Full classifiers relevant coefficients are provided at Data S1.

The coefficients of the *TP53* model should be interpreted with care, for several reasons: some of these genes may change in expression levels via indirect association meaning they may not be directly regulated by *TP53*; the gene set may omit genes that are *bona fide TP53* targets if the information contained in them is redundant with other genes; and finally these genes may individually be only weakly associated with *TP53* status, since the method optimizes the expression markers’ collective power. Visualization was performed using Revigo [78].

#### *TP53* status detection in cell lines

Using the downloaded RNAseq from GDSC cell lines data we applied our trained tumor classifier to cell lines. As stated above, RNAseq data was square rooted, normalized and ComBat batch corrected. Cell line prediction performance was measured using as reference *TP53* COSMIC labels [79] combined with Iorio et al. methodology [73] as we did in tumors. FDR was calculated again using samples harboring an amplification of known phenocopying genes (*MDM2*, *MDM4*, *PPM1D*) or *TP53* deletions as true positives.

Using the classifier scores we separate the cell lines high-confidence set (FDR <  = 18%) using as threshold reference GISTIC tresholded *TP53* deep deletions (-2) (threshold = 0.93) (FirebrowseR package, Analyses.CopyNumber.Genes.Thresholded function). Therefore, we determine 3 sets derived from our Phenocopy score: high-confidence set (predicted *TP53* phenocopies, classified as mutant but originally labeled as wild type), *TP53* mutant (classified and labeled as mutant) and *TP53* wild type (classified and labeled as wild type). All cell line predictions are provided at Data S3.

Due to a lack of positive controls, samples that were classified as wild type being originally labeled as *TP53* mutant were not considered further. However, in the future, analyses with a higher number of cancer genomes may reveal mechanisms of re-establishing *TP53* activity in some *TP53* mutant cancers (e.g. by normalizing expression of the *TP53*-downstream genes which have been dysregulated by the *TP53* mutation).

#### Gene codependency with *TP53* knockout/knockdown in cell line screens

Following data of the 2021 Q4 release downloaded from the DepMap project website: CRISPR data from PROJECT Score [26] (“Achilles_gene_effect.csv”), combined RNAi from DEMETER2 scores repository [27] (“D2_combined_gene_dep_scores.csv”), and the cell line metadata (“sample_info.csv”). In this data, negative scores imply cell growth inhibition and/or death following gene knockout.

CRISPR data is normalized so non-essential genes scores are close to 0. We used Pearson's correlation to correlate the gene effect of CRISPR *TP53* knockout in every cell line to other genes' effect. We tested 990 cell lines for 12,419 genes. This correlation score was calculated both pan-cancer and by cancer type.

Similarly as with CRISPR codependency data we correlated gene knockdown effect with *TP53* knockdown (RNAi) effect using Pearson's correlation test. We tested 700 cell lines for our 12,419 genes. This score was calculated both for pan-cancer and by cancer type.

#### Calculation of the combined gene prioritization score

We sought to rank possible *TP53* loss phenocopying genes testing different data: copy number variant data, gene expression data (RNAseq), RNAi codependency score and CRISPR codependency score. We used the downloaded tumor data (previously described) and our *TP53* Phenocopy score to test for differences across our 3 main *TP53* groups: *TP53* wild type (labeled and classified as wild type), *TP53* mutated (labeled and classified as mutated) and predicted *TP53* phenocopied (labeled as wild type but classified as mutated). We guessed that phenocopying genes should have a differential expression in the phenocopies group when comparing to wild type and mutated *TP53* groups individually. We tested 12,419 genes (by cancer type) in the following manner (via Student's t-test):

- CNV_gene_*TP53*_wt *versus* CNV_gene_*TP53*_phenocopies (CNV0 test),
- CNV_gene_*TP53*_mut *versus* CNV_gene_*TP53*_phenocopies (CNV1 test)
- GE_gene_*TP53*_wt *versus* GE_gene_*TP53*_phenocopies (GE0 test)
- GE_gene_*TP53*_mut *versus* GE_gene_*TP53*_phenocopies (GE1 test)
- RNAi_score_gene *versus* RNAi_score_TP53 (RNAi codependency score, methodology described above)
- CRISPR_score_gene *versus* CRISPR_score_TP53 (CRISPR codependency score, methodology described above).

Three thousand ten genes did not have gene expression data so GE1 and GE0 tests were omitted from the combination test for those genes. We combined the *p*-values values from all available tests by cancer type using Fisher's method for combining *p*-values. For each category (CNV and GE) we only use in the combination the worst *p*-value (i.e. maximum) between CNV0 and CNV1 test, and separately between GE1 and GE0 test, as a way of aggregating. Genes in which the test direction is not coherent in CNV, GE and codependency score were dropped. A gene with a negative codependency score, such as a negative regulator such as *MDM2*, is expected to cause a phenocopy of *TP53* by amplification and overexpression (therefore a higher expression in the phenocopies group that *TP53* wt or mut). *P*-values were FDR adjusted using Benjamini–Hochberg method (p.adjust function of the stats R package). We further merged each cancer type combined *p*-values into one single *p*- value using Fisher's approach, and FDR adjusted. That way we obtained the final Prioritization score for each gene in a cancer-combined way. Pearson R score of CRISPR/RNAi codependency was merged across cancer types by taking its mean value.

We set as reference points the known phenocopying genes’ (*MDM2*, *MDM4*, and *PPM1D*) FDRs and CRISPR codependency scores. To establish a stringent threshold for new candidate phenocopying genes, we required that a gene's prioritization score should have an FDR as significant as the best-ranked known phenocopying gene (considered by cancer type). Same requirement was applied for the CRISPR codependency score. For example, the known phenocopying genes with the best score by cancer type was *MDM4* in the LUAD cancer type, with an combined FDR of 4e-05 and a CRISPR codependency score of -0.21. Finally, we additionally applied a pan-cancer CRISPR and RNAi codependency score threshold, requiring genes to have at least a modest correlation (absolute value >  = 0.1) between the TP53 knockout/knockdown fitness a candidate gene knockout fitness in both CRISPR and RNAi screens (codependency score); this filter removes many of the passenger neighboring genes that are co-amplified with known phenocopying genes.

#### *TP53* wild-type and *TP53* -/- isogenic cell line genetic screens

Mean beta scores were calculated using MAGeCK-MLE [80] for *TP53*-isogenic pair cell lines A549 [81] and two RPE1 cell lines [82, 83]. Beta scores represent the effect that gene knock-out has on cell fitness.

We calculated the Z-scores (distance from the mean expressed as number of standard deviations) of either *USP28* or ATM within the distribution of their respective neighbor genes, for each dataset and *TP53* status "1Mbp neighbor genes" are genes present in Brunello [84] and Gecko v2 [85] libraries and located within a 1Mbp window surrounding either *USP28* or ATM, obtained from genecards.weizmann.ac.il.

#### Drug response associations with *TP53* status

We collected GDSC [73] drug data for a total of 1000 cell lines. We used IC50 as a measure of activity of a compound against a specific cell line. If drug data was available in both GDSC1 and GDSC2 versions, GDSC1 data was selected.

We also collected each drug putative target and target pathway information from the GDSC website (https://www.cancerrxgene.org/). We filtered out NA values and transformed IC50 scores to log scale. We downloaded GDSC mutational Cancer Functional Events (CFEs) [73] in order to: make comparisons between *TP53* Phenocopy score and GDSC *TP53* CFEs and to test other gene status-drug response associations, while controlling for *TP53* status. Mutational CFEs consist of a GDSC curated set of cancer genes (CGs) for which the mutation pattern in whole-exome sequencing (WES) data is consistent with positive selection.

We first used drug response (IC50) values of 449 GDSC drugs to fit a pan-cancer regressions against *TP53* status using cancer type as control variable. We fit three different regressions per drug response: against *TP53* CFEs, against predicted *TP53* Phenocopy thresholded scores and against *TP53* random labels.

$$log\mathit(IC\mathit{50}\mathit)\mathit\sim TP\mathit{53}\mathit.status\mathit+cancer\mathit.type$$

For the *TP53* status we used the groups obtained from our phenocopy score being the *TP53* high-confidence phenocopying set (classified as mutant by the score, but labeled as *wild-type* genetically) and *TP53* mutant set (classified as mutant, labeled as mutant) the *TP53* deficient set (*TP53*.status = 1) and *TP53* wild type (classified as *wild-type*, labeled as *wild-type*) as wild type set (*TP53*.status = 0). Due to uncertainty, we filtered out samples with a *TP53* mutation classified as *wild-type*. Cancer types with less than 3 cases for any category were filtered out. We used the *esc* R package to calculate effect size (cohens_d function). *P*-values of associations were FDR corrected using the Benjamini-Hochberg (“fdr”) correction of the p.adjust function (stats package).

We separate the drugs into groups according to the pathway the gene they target belong to. By pathway, we calculated the slope resulting from the comparison of the FDR phenocopy score regression versus the FDR *TP53* CFEs. For the visualization we plotted raw IC50 values of different drugs and all drugs together across the different cell line defined sets. For further analysis, we merged the cancer types that are thought to have some similarity: HNSC with LUSC (jointly known as HNSC_LUSC), GBM with LGG (LGG_GBM) and OV with UCEC (OV_UCEC).

#### Drug response associations of gene status controlling for *TP53* status

We collected drug screening data from the PRISM project [52] and GDSC project [51]. NA values were filtered out and IC50 values were transformed to logarithmic scale. We downloaded mutation features (GDSC mutational CFEs, see above) from [73]. 

First, we fit a regression for each drug and gene CFE including *TP53* loss Phenocopy score and the interaction term as it follows:

$$log\mathit(IC\mathit{50}\mathit)\mathit\sim genCFEs\mathit+TP\mathit{53}Phenocopy\mathit.status\mathit+genCFEs\mathit\ast TP\mathit{53}Phenocopy\mathit.status$$

For comparison, we performed the same analysis using *TP53* random and *TP53* CFEs instead of *TP53* Phenocopy.status.

We tested every gene mutational CFEs out of the 300 genes provided by GDSC. We filtered out cases with lss than 3 samples in any category (mutated:1 or wildtype:0) for *TP53* status and gen CFEs. Regressions were fitted by cancer type using *glm* package (glment 4.0–2 R package). We selected genCFEs *p*.value and FDR correct using the Benjamini-Hochberg (“fdr”) correction of the p.adjust function (stats R package). The coefficient of the genCFEs variable informs us about the fold change of the different variable states (mutant:1-wildtype:0) when *TP53*Phenocopy.status is set to its reference levels (wildtype:0). We compared these scores when using *TP53* Phenocopy to *TP53* CFEs by plotting FDR values and calculating slope (Fig. 5a, Additional file 1: Fig. S10a).

#### Two-way association tests for gene-drug associations modified by TP53

To further analyze *TP53* interaction in a more stringent way we implemented a version of the “two-way association test” approach recently developed by us and reported in Levatic et al. [55]. In this methodology we enforced that, for a given drug, an association between a GDSC gene feature (e.g. gene mutation CFEs) and GDSC drug response is reproduced in other drugs with the same molecular target; here this is additionally controlled by *TP53* status.

For this, we curated 996 sets of two drugs with the same target (ie: Dabrafenib and AZ628, target = BRAF). For the two drugs separately, we fitted a regression comparing the GDSC drug response against gene mutational status (CFE) controlling for *TP53* status (as stated above) by cancer type. We tested the different labels in the regression: *TP53* CFEs, *TP53* random labels and *TP53* phenocopy labels. We considered associations by cancer type. We calculated the two-way association score by averaging the estimates (effect size) obtained between drug 1 and drug 2. To calculate the *p*-value for each drug-drug combination, we shuffled the *TP53* labels and compared the obtained random estimates with the actual estimate as described in our previous work [55].

For an association to be selected, we require that it is observed in more than one cancer type (merged cancer types excluded), FDR < 25% across all cancer types where the hit is observed and that the direction (value from gene CFEs variable estimate) is maintained across drugs. When selecting relevant hits we also required that each hit *TP53* interaction term variable in regression is significant (FDR < 25%). This informs us of deviation from the behavior of the regression variables gen_status = 1 and gen_status = 0 when *TP53* is controlled as interaction. We filtered out cases with less than 3 samples in any category (mutated:1 or wildtype:0) for *TP53* status and gene CFEs in a cancer type-specific manner. Supported hits by this methodology are reported at Fig. 6 b,c, Additional file 1: Fig. S10c, Additional file 1: Fig. S11a and e and in Additional file 4: Data S7.

In addition, as a validation for some hits we performed a “two-way” using PRISM data. In this case we enforced that, for a given drug, an association between a gene feature (GDSC gen mutational CFEs) and GDSC drug response is reproduced in the same drug using the PRISM dataset. The rest of the methodology was applied in the same manner (see GDSC “two-way test” above).

As control, we followed the same procedure of the two-way testing method but fitting regressions of IC50 ~ gene CFEs (without interaction term). FDR corrected *p*-values of gen CFEs coefficient in regressions with and without interaction term were compared. We made different types of comparisons: by gene associations (Additional file 1: Fig. S10b), molecular target-gen CFEs associations (different 2-sets of drugs can target the same molecular feature) and all associations (Additional file 1: Fig. S10a).

## Supplementary Information

**Additional file 1: Supplementary Fig. 1.** Mechanisms related to *TP53* loss phenocopying in tumors. **Supplementary Fig. 2.** Validation of TP53 phenocopy scores. **Supplementary Fig. 3.** Prioritization score methodology. **Supplementary Fig. 4.** Further evidence supporting the role of individual *TP53*-loss phenocopying genes. **Supplementary Fig. 5.** Evidence supporting USP28 as a chromosome 11 phenocopying gene. **Supplementary Fig. 6.** Supporting evidence for effects of *CNOT2* and *MSI2* in the amplified segments bearing the *MDM2* and *PPM1D* genes, respectively. **Supplementary Fig. 7.** Additional data on effects of *TP53* loss and phenocopying thereof on cancer cell line drug sensitivity. **Supplementary Fig. 8.** Pathway-wise effects of *TP53* mutations or phenocopies on drug sensitivity of cancer cell lines. **Supplementary Fig. 9.** Cancer type specific effect of TP53 phenocopies or mutations in drug testing. **Supplementary Fig. 10.** Associations between mutation status of various genes and the sensitivity to various drugs can be modulated by the *TP53* functional status. **Supplementary Fig. 11.** Additional analyses of TP53 status modulating associations between driver mutations and drugs.

**Additional file 2.**

**Additional file 3: Supplementary Data 1.** TCGA TP53 Phenocopy scores. **Supplementary Data 2.** Gene coefficients. **Supplementary Data 3.** PCAWG TP53 Phenocopy scores. **Supplementary Data 4.** Prioritization scores. **Supplementary Data 5.** USP28/ATM fitness effect. **Supplementary Data 6.** Cell lines TP53 Phenocopy score.

**Additional file 4: Supplementary Data S7.** Two-way association tests for drug activity.

## Data Availability

In this study published datasets were reanalyzed. TCGA RNAseq gene expression levels were downloaded from the TCGA repository at NCI Genomic Data Commons [GDC Data Portal https://portal.gdc.cancer.gov/ (2016)]. TCGA CNV data was downloaded using the Firebrowser R package [https://github.com/mariodeng/FirebrowseR] using the Analyses.CopyNumber.Genes.Thresholded function. Tumor purity data was downloaded using the *Tumor.purity* function from TCGAbiolinks 2.25.3 [https://github.com/BioinformaticsFMRP/TCGAbiolinks]. PCAWG RNAseq and CNV were downloaded using the Xena browser [donor centric miRNA expression and copy number,https://pcawg.xenahubs.net]. Cell line RNAseq data was downloaded from CLLE [21Q4 CCLE_expression.csv https://depmap.org/portal/download/all/ (2021)] and GDSC [Expression array, https://www.cancerrxgene.org/downloads/bulk_download (2014)]. Cell line CNV data was downloaded from Depmap [21Q4 CCLE_gene_cn.csv https://depmap.org/portal/download/all/ (2021)].Somatic mutation calls for TCGA were downloaded from the MC3 project [mc3.v0.2.8.PUBLIC.maf.gz https://gdc.cancer.gov/about-data/publications/mc3-2017 (2017)]. TP53 variant pathogenic scores were downloaded from EVE [TP53 EVE score https://evemodel.org/download/protein/P53_HUMAN (2021)] and Varity [gen name: TP53, http://varity.varianteffect.org (2021)]. PCAWG TP53 status was downloaded from Xena [*simple somatic mutation (SNVs and indels) – coding driver mutations*, https://pcawg.xenahubs.net]. Cell line gene mutation data (CFEs) and TP53 mutational CFEs were downloaded from GDSC [Mutational CFEs, https://www.cancerrxgene.org/gdsc1000/GDSC1000_WebResources/Clinically_Relevant_Features.html (2014)]. CRISPR PROJECT score data was downloaded from Depmap [2021Q4 Achillles_gene_effect.csv, 10.6084/m9.figshare.16924132.v1 (2021)]. DEMETER2 RNAi scores were downloaded from repository project [D2_combined_gene_dep_scores.csv, DEMETER2 data 10.6084/m9.figshare.6025238.v6 (2018)]. Drug data, drug putative target and target pathway information from the GDSC website [Drug Screening – IC50s, https://www.cancerrxgene.org/downloads/bulk_download (2014)] and from PRISM project [PRISM data 10.6084/m9.figshare.20564034.v1 (2022)].

## Abbreviations

AUROC: Area under the receiver operating characteristic
LOCTO: Leave-one-cancer-type-out
AUC: Area under the curve
AEN: Apoptosis-enhancing nuclease
CFE: Cancer Functional Events
CNA: Copy number alteration
FDR: False discovery rate
LoF: Loss-of-function
ROC: Receiver operating characteristic
TPM: Transcripts per million
