# Supplementary material for: Prevalence, causes and impact of TP53-loss phenocopying events in human tumors
Source: BMC Biol. 2023 Apr 24;21:92. doi: 10.1186/s12915-023-01595-1 (PMC10127307; doi:10.1186/s12915-023-01595-1)
Supplement: Supplementary file 1 — Additional file 1: Supplementary Fig. 1. Mechanisms related to TP53 loss phenocopying in tumors. Supplementary Fig. 2. Validation of TP53 phenocopy scores. Supplementary Fig. 3. Prioritization score methodology. Supplementary Fig. 4. Further evidence supporting the role of individual TP53-loss phenocopying genes. Supplementary Fig. 5. Evidence supporting USP28 as a chromosome 11 phenocopying gene. Supplementary Fig. 6. Supporting evidence for effects of CNOT2 and MSI2 in the amplified segments bearing the MDM2 and PPM1D genes, respectively. Supplementary Fig. 7. Additional data on effects of TP53 loss and phenocopying thereof on cancer cell line drug sensitivity. Supplementary Fig. 8. Pathway-wise effects of TP53 mutations or phenocopies on drug sensitivity of cancer cell lines. Supplementary Fig. 9. Cancer type specific effect of TP53 phenocopies or mutations in drug testing. Supplementary Fig. 10. Associations between mutation status of various genes and the sensitivity to various drugs can be modulated by the TP53 functional status. Supplementary Fig. 11. Additional analyses of TP53 status modulating associations between driver mutations and drugs. [file 12915_2023_1595_MOESM1_ESM.pdf]

Additional File 1. Supplementary Figures for Fito-López *et al*. (2023) BMC Biology.

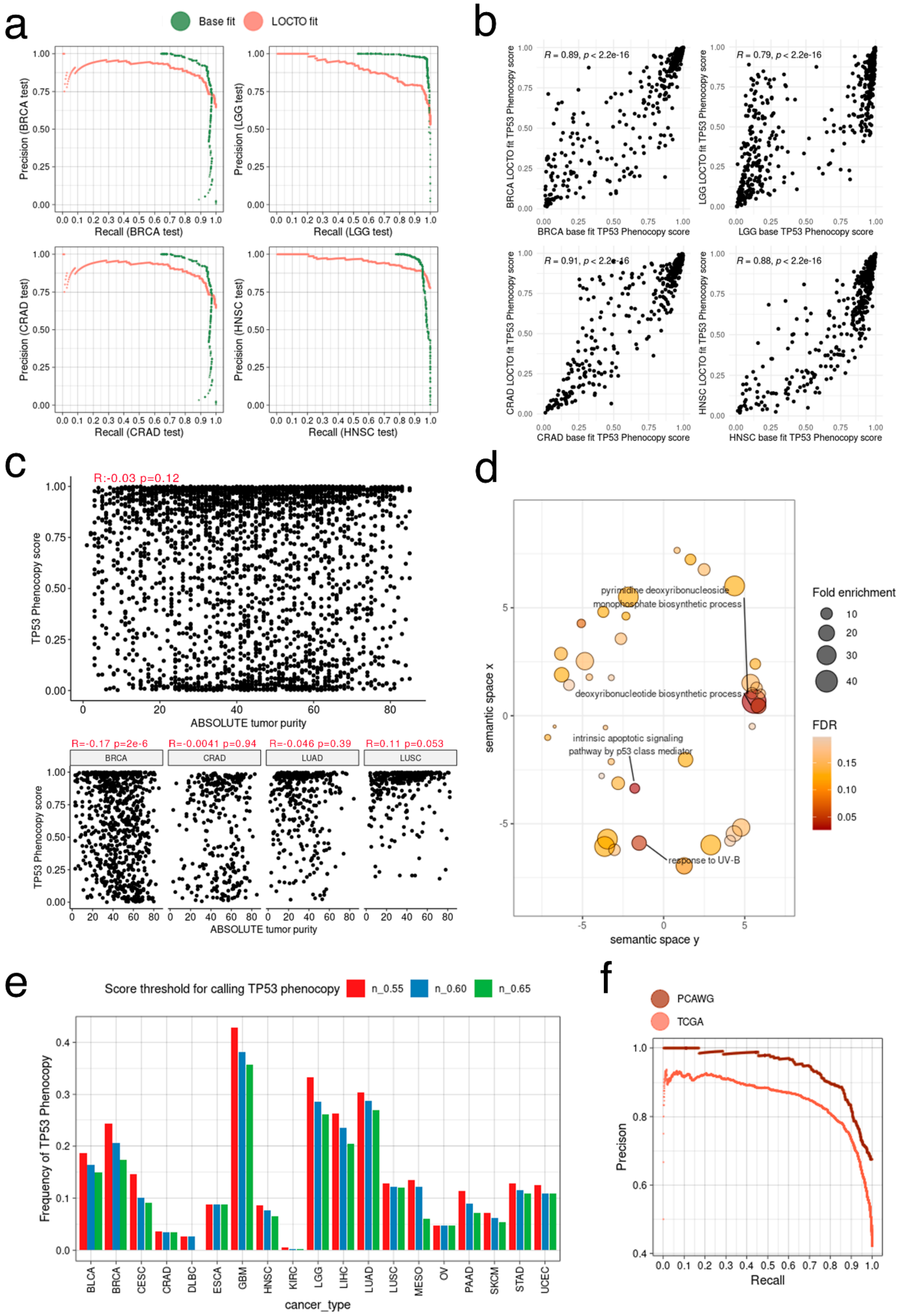

Supplementary Figure 1. Mechanisms related to *TP53* loss phenocopying in tumors.

- **a)** Precision-recall curves for LOCTO classifier and baseline classifier. Curves show precision vs recall for LOCTO classifiers trained excluding samples of a particular cancer type (panels), vs a baseline classifier trained on all samples. Each panel represents a cancer type that was used for testing: the corresponding LOCTO classifier or the base classifier with all samples. Each dot represents a sample. X axis represents the recall and Y axis represents precision (using as threshold each sample classifier's probability).
- **b)** TP53 phenocopy score (classifier probabilities) of LOCTO and baseline classifier. Top: Each dot shows assigned probabilities of LOCTO classifiers (Y axis) trained excluding samples of a given cancer type (panels), vs a baseline classifier´s probabilities trained using all samples (X axis). Pearson R score is displayed for each cancer type. Bottom: same but stratified by cancer type
- **c)** Tumor purity correlation with TP53 phenocopy score. X axis represents tumor purity obtained by ABSOLUTE algorithm (see Methods). Y axis represents the TP53 phenocopy score obtained by our baseline TCGA classifier. Each dot represents a tumor sample. Pearson R score is displayed.
- **d)** Revigo visualization of the Gene Ontology (GO) enrichment of the *TP53* transcriptomic classifier coefficients. The axes have no intrinsic meaning, and semantically similar GO terms should remain close together in the plot (see REVIGO publication for details (27). Size indicates fold enrichment of a given GO functional category in the set of genes that had non-zero coefficients in the *TP53* classifier, while color indicates FDR of the GO enrichment.
- **e)** TP53 phenocopies across different thresholds. Each color represents the threshold used for calling the frequency of phenocopies (Yaxis) across cancer types (X axis)
- **f)** Precision-recall curves for PCAWG classifier and TCGA classifier. Curves show precision vs recall for the original TCGA classifier and PCAWG, our external validation classifier. Each dot represents a sample. X axis represents the recall and Y axis represents precision (using as threshold each sample classifier's probability).

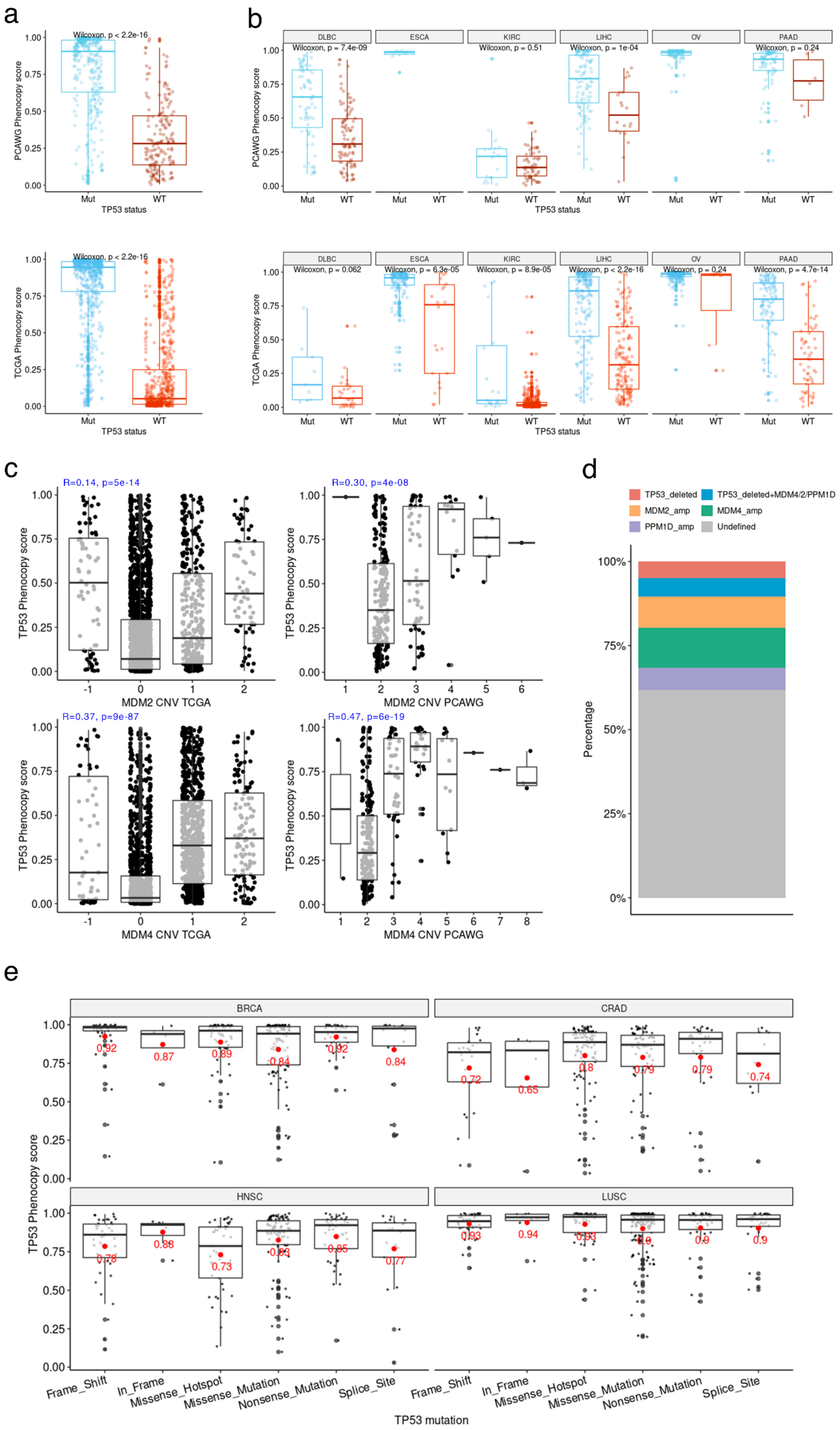
a
b
c
d
e
Wilcoxon, p < 2.2e-16
PCAWG Phenocopy score
TCGA Phenocopy score
Mut
WT
TP53 status
DLBC
ESCA
KIRC
LIHC
OV
PAAD
Wilcoxon, p = 7.4e-09
Wilcoxon, p = 0.51
Wilcoxon, p = 1e-04
Wilcoxon, p = 0.24
Wilcoxon, p = 0.062
Wilcoxon, p = 6.3e-05
Wilcoxon, p = 8.9e-05
Wilcoxon, p < 2.2e-16
Wilcoxon, p = 0.24
Wilcoxon, p = 4.7e-14
R=0.14, p=5e-14
R=0.30, p=4e-08
R=0.37, p=9e-87
R=0.47, p=6e-19
TP53 Phenocopy score
MDM2 CNV TCGA
MDM2 CNV PCAWG
MDM4 CNV TCGA
MDM4 CNV PCAWG
TP53_deleted
TP53_deleted+MDM4/2/PPM1D
MDM2_amp
MDM4_amp
PPM1D_amp
Undefined
Percentage
BRCA
CRAD
HNSC
LUSC
Frame_Shift
In_Frame
Missense_Hotspot
Missense_Mutation
Nonsense_Mutation
Splice_Site
TP53 mutation

Supplementary Figure 2. Validation of TP53 phenocopy scores.

a) Comparison of TP53 phenocopy score across TP53 status. Top plot represents phenocopy scores in PCAWG dataset. Bottom represents the TCGA dataset.Wilcoxon test p values from the comparison of TP53 WT vs TP53 Mut are displayed on each plot. The TP53 Mut group represents samples with a TP53 mutation or a TP53 deep deletion.

b) Similar to C, but stratified according to the common cancer pairs of the TCGA-PCAWG dataset. Top is for PCAWG, bottom is for TCGA. Cancer types in PCAWG are named under the equivalent TCGA abbreviation:ESAD/ESCA,CLLE/DLBC,MALY/DLBC,RECA/KIRC,PACA/PAAD, LIRI/LIHC

c) TP53 phenocopy score across known phenocopying events (MDM2/4). Right plots show PCAWG phenocopy score, left plot show TCGA phenocopy scores. X axis represent thresholded CNV data from GISTIC2 (left) and PCAWG-11 consensus CNV (right). TP53 mutated/deleted samples were excluded. Pearson correlation test is shown on top of each plot.

d) Similar to Fig.1A, however the subset of *TP53* loss phenocopying tumor samples was further stratified into tumors with known phenocopying CNA events (colors), or the majority in the "Undefined" category (accounts for cases in which the cause of the phenocopy is unknown).

e) TP53 phenocopy score for TP53 mutations. Top 4 cancer types with more TP53 mutated samples are shown (panels). Each boxplot represents a category of TP53 mutation with its mean plotted (red dot and text). Each dot represents a sample.

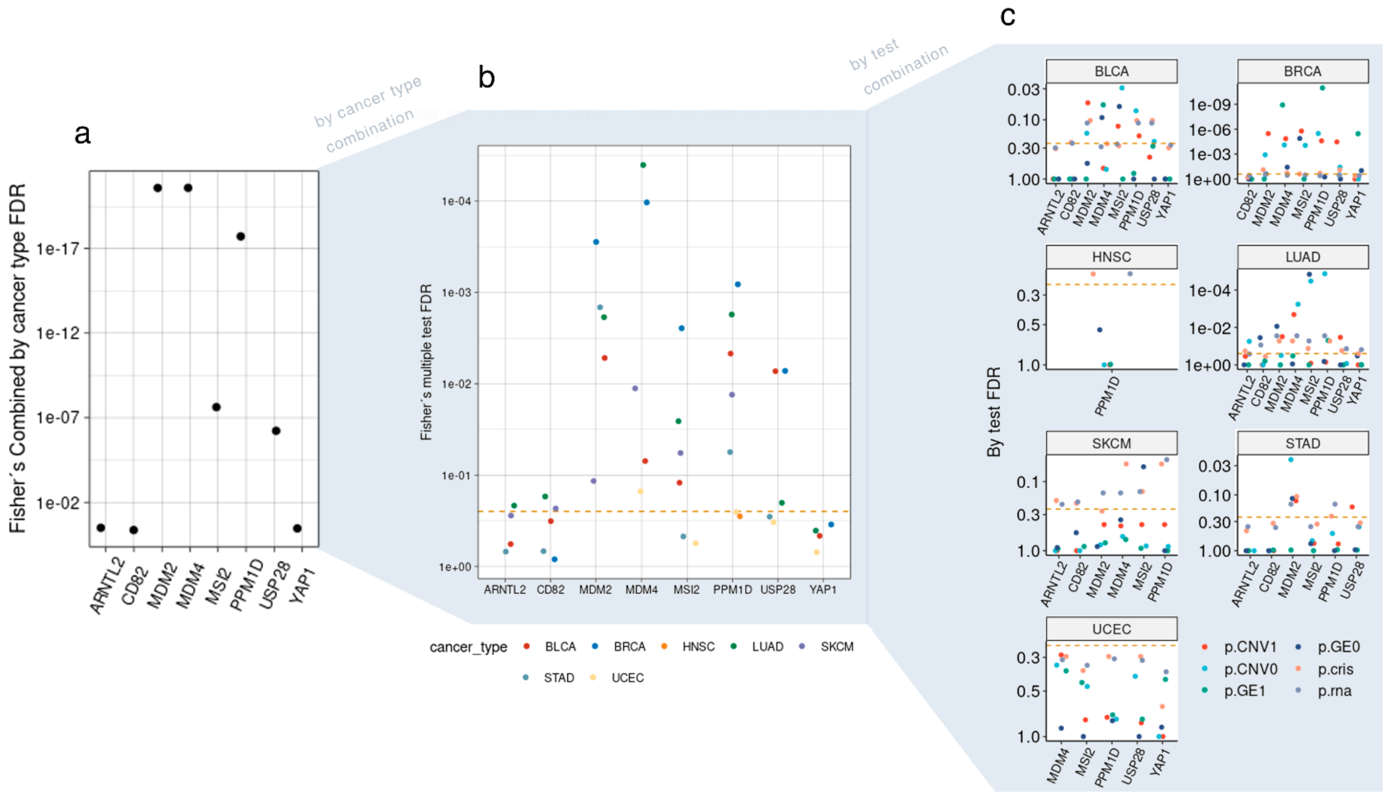

Supplementary Figure 3. Prioritization score methodology

a) Prioritization score of top genes merged across cancer types. Each score combines (Fisher's method for combining p-values) six tests across four different data types (gene expression association to phenocopied samples over rest [GE], CNV association to phenocopied samples over rest phenocopy score [CNV], CRISPR *TP53* codependency, RNAi *TP53* codependency, see Methods) for each gene considered. The p-values across cancer types scores are also merged together, and final p-values are FDR corrected. The Y axis represents the final prioritization FDR of each gene. X axis represents a set of top genes, and additionally three random genes as control (*CD82, YAP1* and *ARNTL2*).

b) Prioritization score p-values (FDR adjusted) stratified by cancer type. Dashed line represents 25% FDR.

c) Prioritization score p-values (FDR adjusted), broken down by variable tested (colors) and by cancer type (panels). Tests compare the score of a phenocopying group of tumor samples versus the rest of tumor groups. Six different statistical tests are considered across four different data types: “pCNV1” (CNV data, versus *TP53* mutated), “pCNV0” (CNV data, versus *TP53* wild-type), “pGE1” (GE data, versus *TP53* mutated), “pGE0” (GE data, versus *TP53* wild-type), “pcris” (CRISPR codependency p-value by cancer type) and “prna” (RNAi codependency p-value by cancer type).

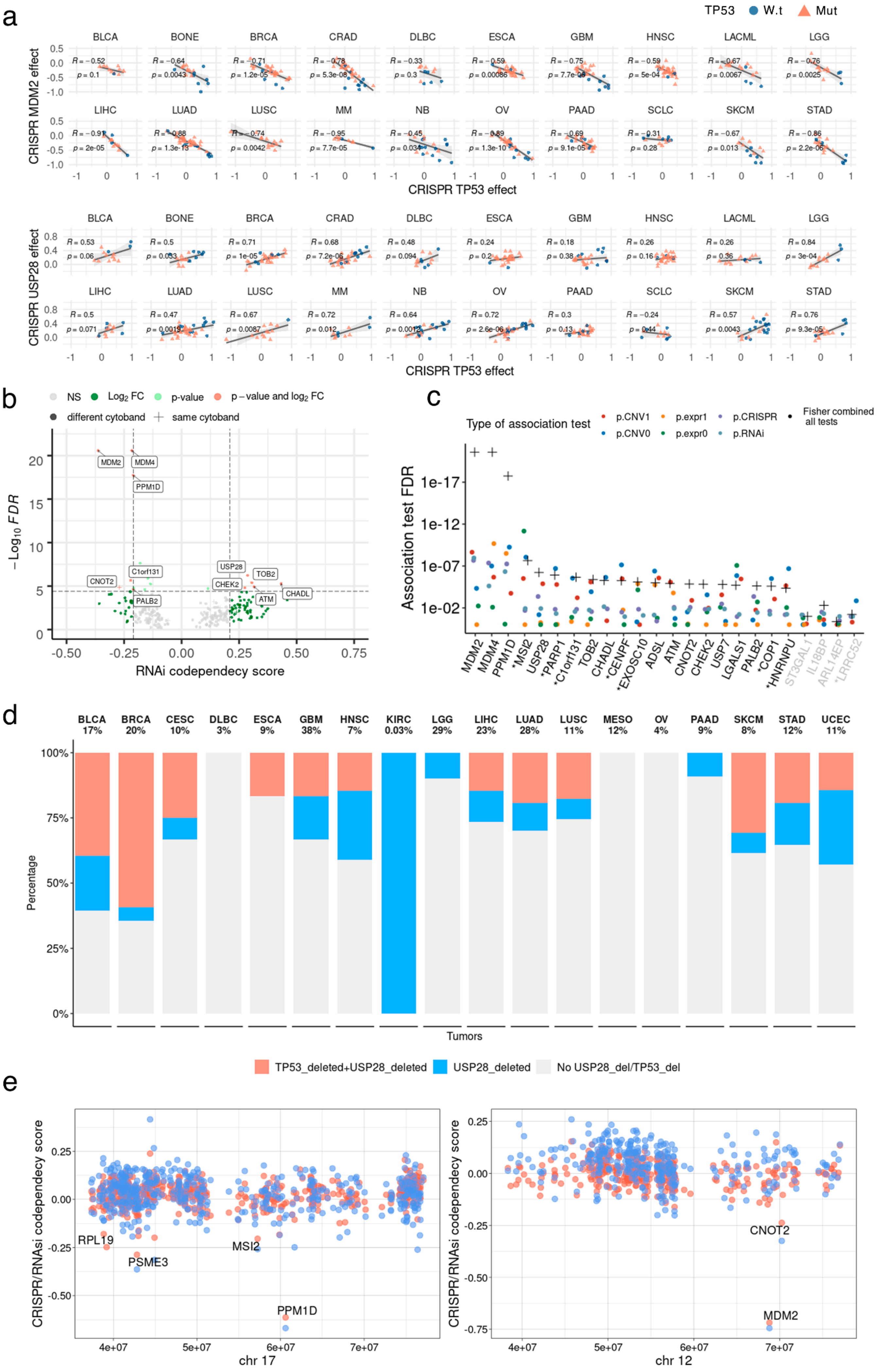
a
TP53 W.t Mut
BLCA BONE BRCA CRAD DLBC ESCA GBM HNSC LACML LGG
LIHC LUAD LUSC MM NB OV PAAD SCLC SKCM STAD
CRISPR MDM2 effect
CRISPR TP53 effect
CRISPR USP28 effect
CRISPR TP53 effect
b
NS Log2 FC p-value p−value and log2 FC
different cytoband same cytoband
MDM2 MDM4 PPM1D C1orf131 CNOT2 PALB2 USP28 CHEK2 TOB2 ATM CHADL
−Log10 FDR
RNAi codependecy score
c
Type of association test
p.CNV1 p.expr1 p.CRISPR Fisher combined all tests
p.CNV0 p.expr0 p.RNAi
Association test FDR
MDM2 MDM4 PPM1D *MSI2 USP28 *PARP1 *C1orf131 TOB2 CHADL *CENPF *EXOSC10 ADSL ATM CNOT2 CHEK2 USP7 LGALS1 PALB2 *COP1 *HNRNPU ST3GAL1 IL18BP ARL14EP *LRRC52
d
BLCA 17% BRCA 20% CESC 10% DLBC 3% ESCA 9% GBM 38% HNSC 7% KIRC 0.03% LGG 29% LIHC 23% LUAD 28% LUSC 11% MESO 12% OV 4% PAAD 9% SKCM 8% STAD 12% UCEC 11%
Percentage
Tumors
TP53_deleted+USP28_deleted USP28_deleted No USP28_del/TP53_del
e
CRISPR/RNAsi codependecy score
RPL19 PSME3 MSI2 PPM1D
chr 17
CNOT2 MDM2
chr 12

Supplementary Figure 4. Further evidence supporting the role of individual *TP53*-loss phenocopying genes.

a) CRISPR screening codependecy scores for *MDM2* and *USP28*. By-cancer type (panel) correlation of TP53 knockout (X axis) and *MDM2*/*USP28* knockout (Y axis). Colors represent different TP53 status for each cell line (dots). Pearson R values are displayed.

b) As Fig. 2A, but showing RNAi screening *TP53* codependency score on the X axis for each gene. Black colored labels are both supported by RNAi score (here) and by CRISPR score (Fig. 2A).

c) Simplified overview of top genes by our prioritization score. The multiple test approach (prioritization test) is broken down by each of the 6 tests (colored dots) that conform it. The final prioritization score ("Fisher combined all test") results from the Fisher combination of each test by cancer type (black cross). Y axis represents the FDR of each test. X axis represents top 20 genes with a higher prioritization score ("Fisher combined all tests'"). Grey colored genes represent a subset of random genes used as negative control. *: gene in the same chromosome arm as MMD2/MDM4/PPM1D. Data is provided at Supplementary Data 4.

d) USP28 phenocopies across cancer types. We considered the "Unknown cause" category (samples without a *MDM2/MDM4/PPM1D* amplification, nor a *TP53* deletion/mutation, but a TP53 phenocopy score >0.6, Fig. 1D) in order to evaluate how many phenocopies would be explained by an USP28 deletion in a cancer specific manner (X axis). Colors represent: samples without any known phenocopying cause, nor a USP28 deletion or *USP28* deletion + *TP53* deletion (grey), *USP28* deleted (blue) and *USP28* deleted + *TP53* deletion (red).

e) CRISPR and RNAi *TP53* codependency scores of segments of chromosome 12 and 17. Genes with a score lower than -0.1 are labeled, highlighting the gene *MSI2* nearby the known phenocopying gene *PPM1D*, and the gene *CNOT2* nearby the known phenocopying gene *MDM2*.

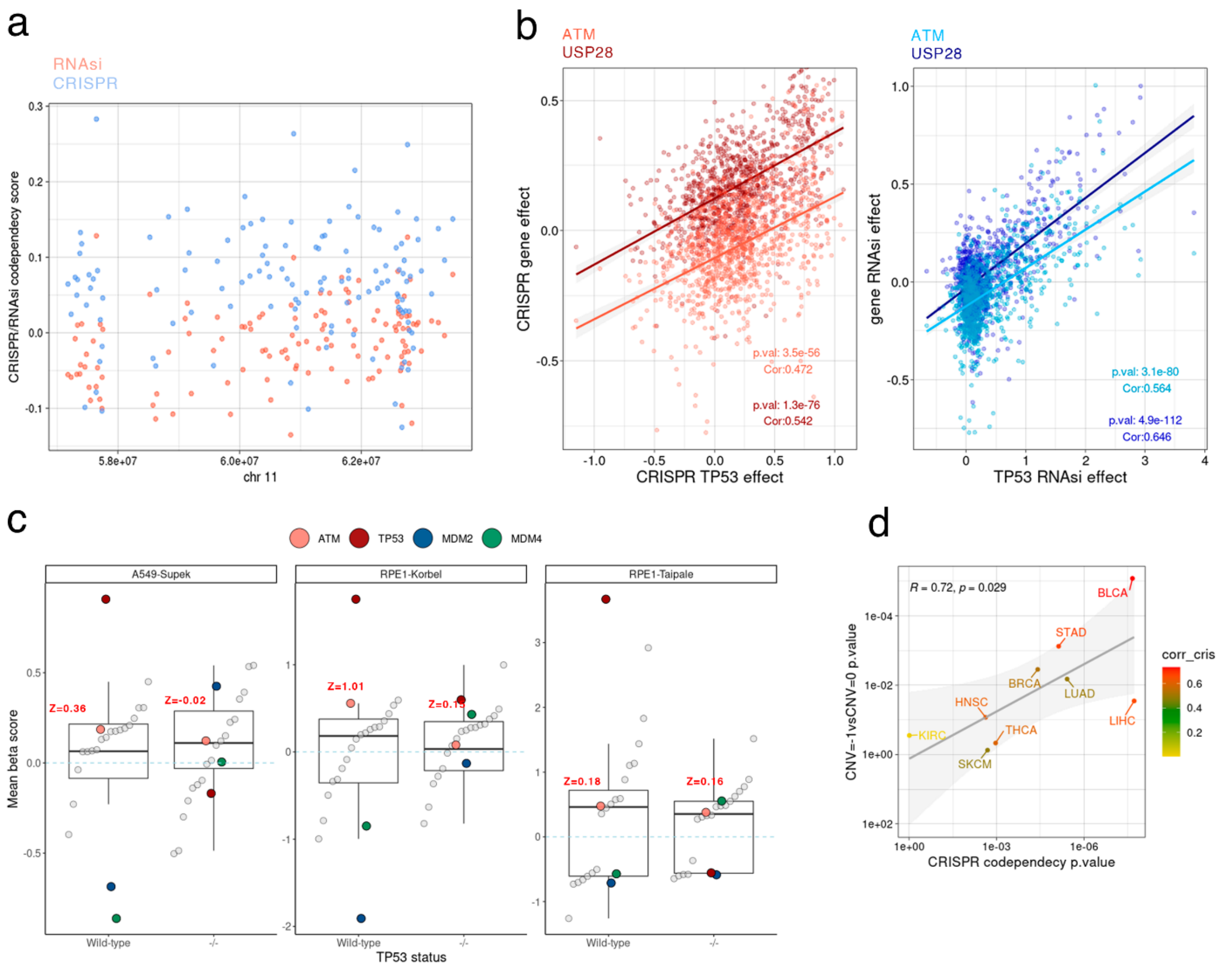

Supplementary Figure 5. Evidence supporting USP28 as a chromosome 11 phenocopying gene

- **a)** CRISPR and RNAi *TP53* codependency scores at chromosome 11 q12.1-q13.2 region. We found a moderate enrichment of CNV deletions in this region when comparing *TP53* loss phenocopied tumor samples against *TP53* wild-type and *TP53* mutant samples. We sought to determine whether for any genes CRISPR and RNAi *TP53* codependency scores were prominent, by analogy to *USP28* codependency scores in the q22-q23 segment.
- **b)** A comparison of the *TP53* codependency for CRISPR fitness effect across many cell lines (left) and RNAi fitness effect (right), between *USP28* and the neighboring ATM gene.
- **c)** Comparison of the mean beta score (fitness effect of CRISPR gene disruption, averaged across replicates; y-axis) of *ATM* with the mean beta scores of genes located within its 1 Mbp immediate surroundings ("1 Mbp neighbors", see Methods). Genes *TP53*, *MDM2*, and *MDM4* are also shown as a reference. x-axis bottom labels indicate the *TP53* status of the cell line. *ATM* Z-scores, comparing to the distribution of neighboring genes, are plotted in red (see Methods)
- **d)** Correlation of CRISPR screen *TP53* codependency for *USP28* (x axis), and *USP28* deletion *TP53* phenocopy scores (y axis), broken down by cancer type. Y axis represents the p-value resulting from the comparison of *USP28* neutral copy number variant *TP53* phenocopy scores versus deleted *USP28* phenocopy scores. Color represents the Pearson correlation coefficient of the CRISPR codependency.

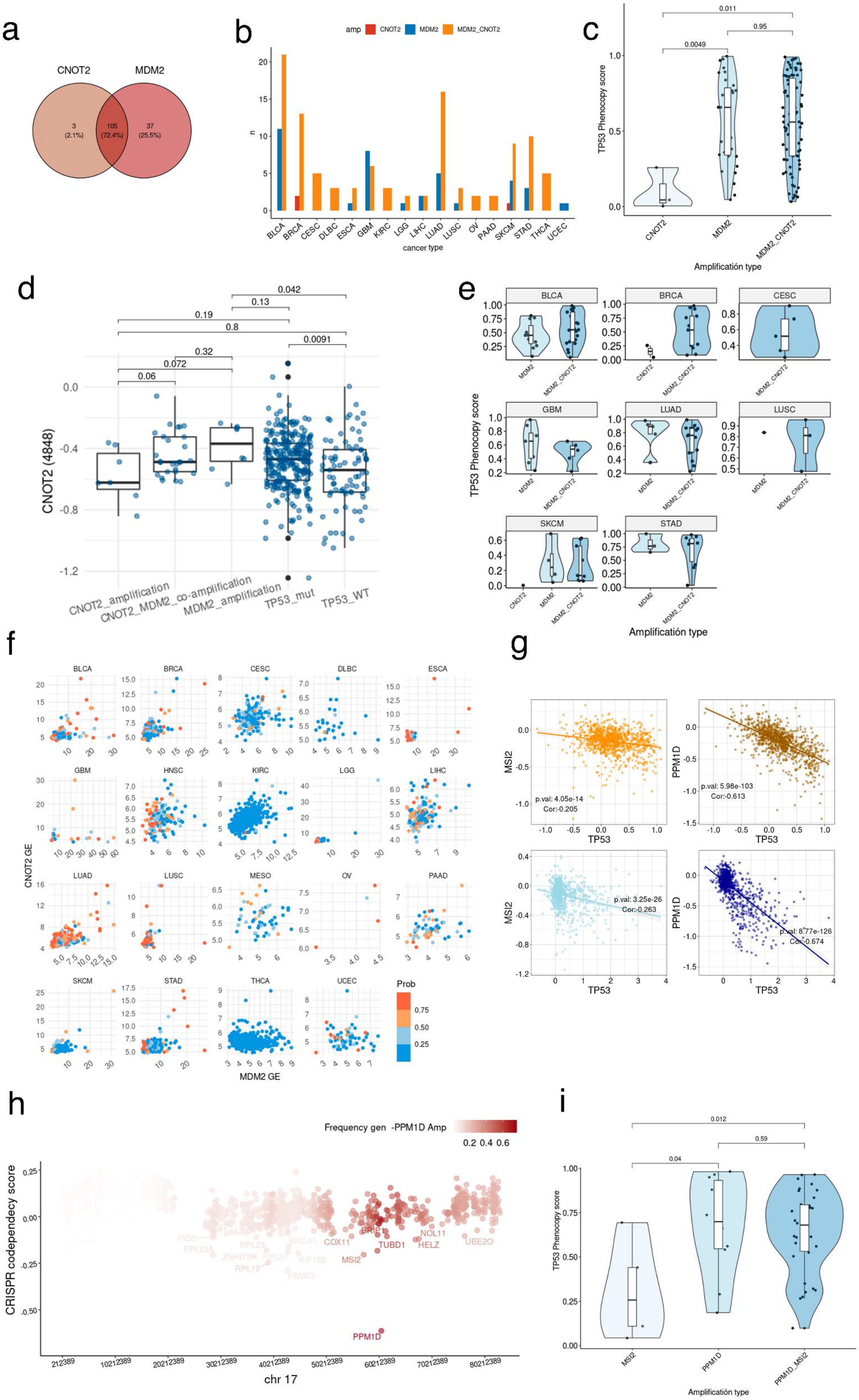
a
CNOT2
MDM2
3 (2.1%)
105 (72.4%)
37 (25.5%)
b
amp CNOT2 MDM2 MDM2_CNOT2
n
cancer type
BLCA BRCA CESC DLBC ESCA GBM KIRC LGG LIHC LUAD LUSC OV PAAD SKCM STAD THCA UCEC
c
TP53 Phenocopy score
Amplification type
CNOT2 MDM2 MDM2_CNOT2
0.011
0.95
0.0049
d
CNOT2 (4848)
0.042
0.13
0.19
0.8
0.0091
0.32
0.072
0.06
CNOT2_amplification
CNOT2_MDM2_co-amplification
MDM2_amplification
TP53_mut
TP53_WT
e
TP53 Phenocopy score
BLCA BRCA CESC GBM LUAD LUSC SKCM STAD
Amplification type
f
BLCA BRCA CESC DLBC ESCA GBM HNSC KIRC LGG LIHC LUAD LUSC MESO OV PAAD SKCM STAD THCA UCEC
CNOT2 GE
MDM2 GE
Prob
0.75
0.50
0.25
g
MSI2
TP53
p.val: 4.05e-14
Cor:-0.205
PPM1D
p.val: 5.98e-103
Cor:-0.613
p.val: 3.25e-26
Cor:-0.263
p.val: 8.77e-126
Cor:-0.674
h
Frequency gen -PPM1D Amp
0.2 0.4 0.6
CRISPR codependecy score
chr 17
PIGS RPL23A ZNHIT3 RPL19 RPL23 RPL27 BRCA1 KIF18B PSME3 COX11 MSI2 BRIP1 TUBD1 NOL11 HELZ UBE2O PPM1D
212389 10212389 20212389 30212389 40212389 50212389 60212389 70212389 80212389
i
TP53 Phenocopy score
Amplification type
MSI2 PPM1D PPM1D_MSI2
0.012
0.59
0.04

Supplementary Figure 6. Supporting evidence for effects of *CNOT2* and *MSI2* in the amplified segments bearing the *MDM2* and *PPM1D* genes, respectively.

- **a)** The number of tumor samples co-amplified for *MDM2* and *CNOT2,* individually for *MDM2* and individually for *CNOT2*.
- **b)** By cancer type *MDM2* - *CNOT2* CNV profiles. X axis represents different cancer types meanwhile Y axis shows the total count of events.
- **c)** Pan-cancer *TP53* phenocopy scores by type of amplification. X axis represents the assigned *TP53* phenocopy score. The Y axis represents the different CNV profiles: *CNOT2* amplifications only, *MDM2* amplifications only and *CNOT2-MDM2* co- amplifications. Cancer types with less than 3 samples were removed. Each dot represents a cell line. P-value of t-test is plotted above each violin plot.
- **d)** *CNOT2* disruption fitness effects suggest genetic interactions with *MDM2* and *TP53*genes. Y axis represents the raw CRISPR score for *CNOT2* knockout (see Methods).X axis is stratified into categories based on the CCLE database CNV states of *MDM2*/*CNOT2* (thresholded at the 90% quantile) and *TP53* mutations (GDSCdatabase CFE labels). Only *TP53 wild-type* samples were considered in every groupexcept “TP53_mut”. Each dot represents a cell line. Mann-Whitney test p-values aregiven above each boxplot.
- **e)** Per cancer type *TP53* phenocopy scores by type of amplification. X axis representsthe assigned *TP53* phenocopy score. The X axis represents the different CNV profiles. Cancer types with less than 3 samples were removed. Each dot representsa cell line.
- **f)** Gene expression (GE) profiles by cancer type. X axis represents *MDM2* gene expression. The Y axis represents *CNOT2* gene expression. Each panel representsa cancer type. Each dot is a cell line. Colors represent the assigned *TP53* phenocopyscores
- **g)** CRISPR and RNAi raw scores for *MSI2* and *PPM1D* Top row represents RNAi codependency scores. Bottom row represents CRISPR codependency score. Rightcolumn shows *PPM1D* RNAi/CRISPR survival score in Y axis and *TP53* RNAi/CRISPR survival score in X axis. Left column shows *MSI2* RNAi/CRISPR survival score in Y axis and *TP53* RNAi/CRISPR survival score in X axis. Correlationcoefficient and p-value is also pictured in each panel.
- **h)** Per-gene chromosome 17 CRISPR codependency scores. Each dot represents a chromosome 17 gene. X axis corresponds to chromosome bp position. Y axis correspond to by gene CRISPR codependency scores. Genes labeled presents a codependency score lower than -0.1. Color shows the frequency of amplification of each gene together with *PPM1D* amplifications.
- **i)** pan-cancer *TP53* phenocopy scores by type of amplification. Y axis represents the assigned *TP53* phenocopy score. The X axis represents the different CNV profiles. Cancer types with less than 3 samples were removed. Each dot represents a cell line. P-value of Fisher's T test is plotted above each violin plot.

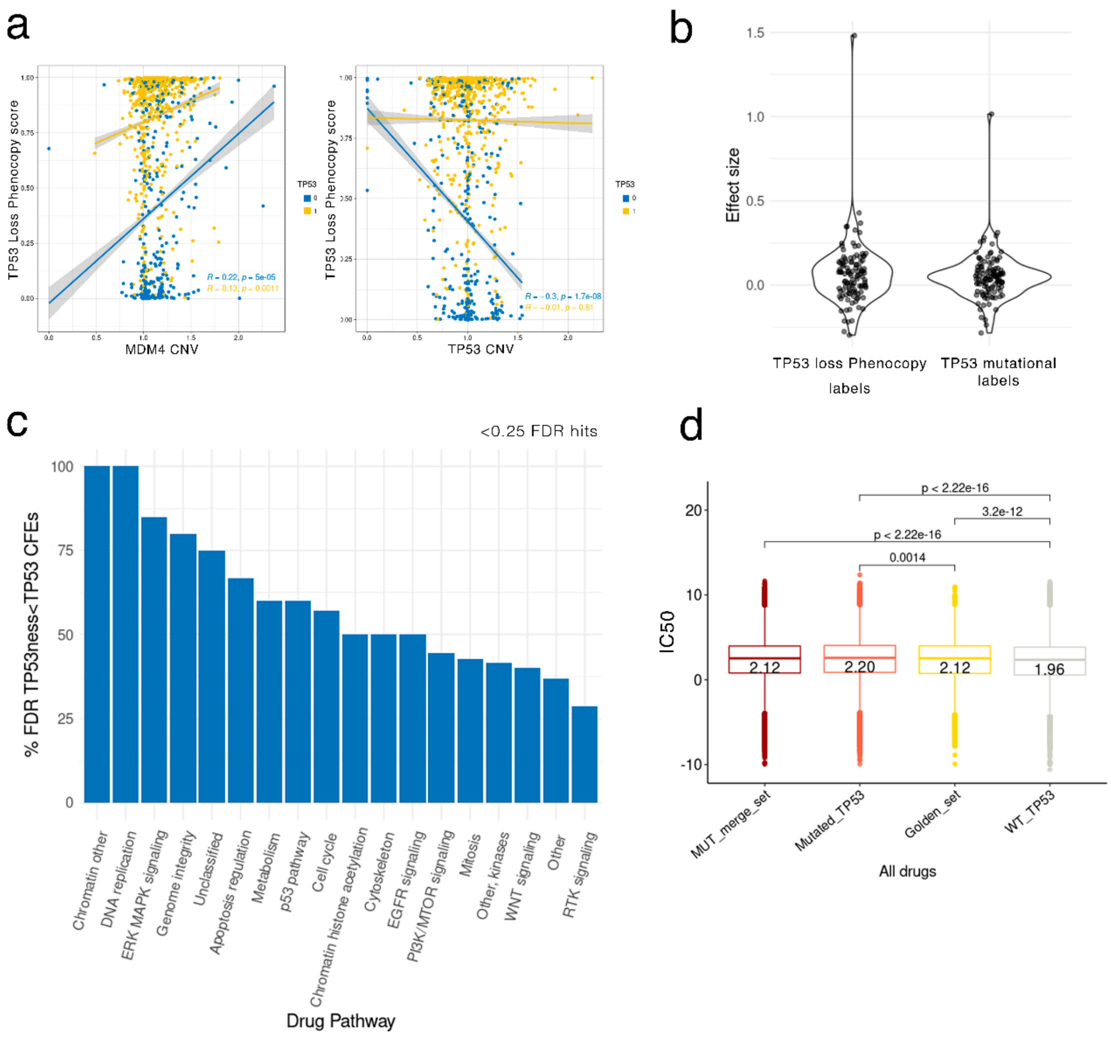

Supplementary Figure 7. Additional data on effects of *TP53* loss and phenocopying thereof on cancer cell line drug sensitivity.

- **a)** *TP53* loss phenocopy score correlation with *MDM4* and *TP53* CNV values. Each dotrepresents a cell line sample colored by *TP53* status, blue for wild type and yellow for mutated. X axis represents the CNV of each given gene. Y axis represents the *TP53* loss phenocopy score obtained by applying the tumor classifier to cell lines. Two regression lines are added in each panel. Pearson's correlation R and p-value of CNV and *TP53* loss phenocopy score is also shown.
- **b)** Effect size for all drugs when fitted with *TP53* status. Y axis represents the effect sizeresulting from fitting a regression [log IC50 ~ *TP53* status] for each drug.
- **c)** Percentage of hits improved when using *TP53* phenocopy score. X axis representsthe different drug categories tested. Y axis represents the frequency of hits in which using *TP53* phenocopy score improved the significance over *TP53* mutational status(CFEs, see Methods). Just hits with an FDR<25% are plotted.
- **d)** Log IC50 values for all drugs in the different confidence categories obtained by our classifier. The X axis illustrates the different categories based on *TP53* mutated status (“Mutated_*TP53*”) and a high *TP53* phenocopy score (high-confidence set or“Golden_set”); the “MUT_merge_set” is a combination of the two. Statistical tests results comparing the groups (Mann-Whitney test, two-sided) are plotted on top. Median values are provided inside of each box.

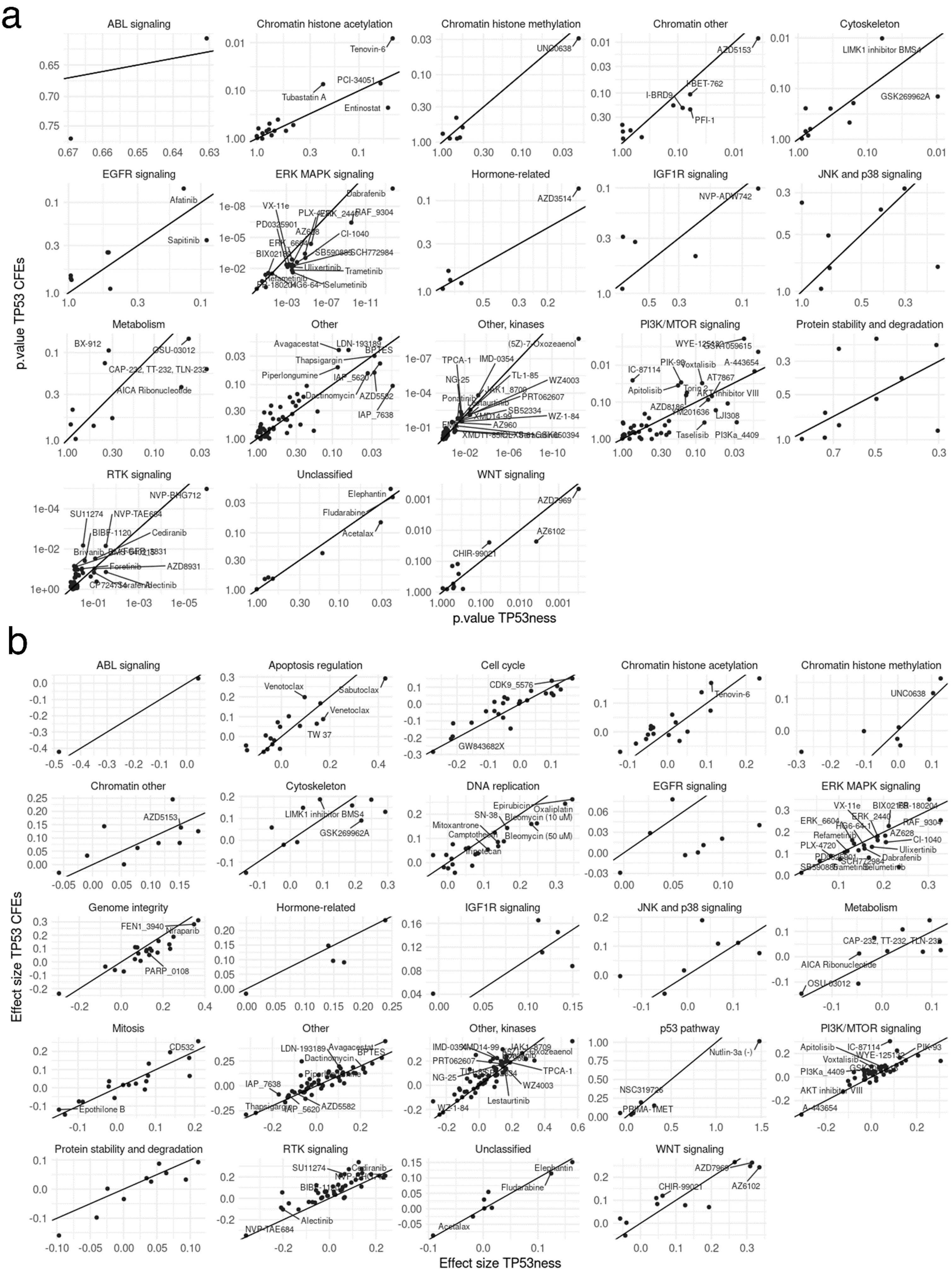
a
ABL signaling
Chromatin histone acetylation
Tenovin-6
PCI-34051
Tubastatin A
Entinostat
Chromatin histone methylation
UNC0638
Chromatin other
AZD5153
I-BET-762
I-BRD9
PFI-1
Cytoskeleton
LIMK1 inhibitor BMS4
GSK269962A
EGFR signaling
Afatinib
Sapitinib
ERK MAPK signaling
Dabrafenib
RAF_9304
CI-1040
Ulixertinib
Trametinib
Refametinib
Selumetinib
Hormone-related
AZD3514
IGF1R signaling
NVP-ADW742
JNK and p38 signaling
Metabolism
BX-912
OSU-03012
CAP-232, TT-232, TLN-232
AICA Ribonucleotide
Other
Avagacestat
LDN-193189
BPTES
Thapsigargin
Piperlongumine
IAP_5620
Dactinomycin
AZD5582
IAP_7638
Other, kinases
(5Z)-7-Oxozeaenol
TPCA-1
IMD-0354
TL-1-85
WZ4003
PRT062607
SB52334
WZ-1-84
AZ960
PI3K/MTOR signaling
GSK1059615
A-443654
IC-87114
PIK-93
Voxtalisib
AT7867
Apitolisib
Torin 2
AKT inhibitor VIII
AZD8186
LJI308
Taselisib
PI3Ka_4409
Protein stability and degradation
RTK signaling
NVP-BHG712
SU11274
NVP-TAE684
BIBF-1120
Cediranib
Brivanib
Foretinib
AZD8931
Alectinib
Unclassified
Elephantin
Fludarabine
Acetalax
WNT signaling
AZD7969
AZ6102
CHIR-99021
p.value TP53 CFEs
p.value TP53ness
b
ABL signaling
Apoptosis regulation
Venotoclax
Sabutoclax
Venetoclax
TW 37
Cell cycle
CDK9_5576
GW843682X
Chromatin histone acetylation
Tenovin-6
Chromatin histone methylation
UNC0638
Chromatin other
AZD5153
Cytoskeleton
LIMK1 inhibitor BMS4
GSK269962A
DNA replication
Epirubicin
Oxaliplatin
SN-38
Bleomycin (10 uM)
Mitoxantrone
Camptothecin
Bleomycin (50 uM)
Irinotecan
EGFR signaling
ERK MAPK signaling
VX-11e
BIX02189
ERK_6604
ERK_2440
RAF_9304
HG6-64-1
AZ628
Refametinib
CI-1040
PLX-4720
Ulixertinib
Dabrafenib
Genome integrity
FEN1_3940
Niraparib
PARP_0108
Hormone-related
IGF1R signaling
JNK and p38 signaling
Metabolism
CAP-232, TT-232, TLN-232
AICA Ribonucleotide
OSU-03012
Mitosis
CD532
Epothilone B
Other
LDN-193189
Avagacestat
Dactinomycin
BPTES
IAP_7638
Thapsigargin
IAP_5620
AZD5582
Other, kinases
IMD-0354
XMD14-99
JAK1_8709
PRT062607
TPCA-1
NG-25
WZ4003
Lestaurtinib
WZ-1-84
p53 pathway
Nutlin-3a (-)
NSC319726
PRIMA-1MET
PI3K/MTOR signaling
Apitolisib
IC-87114
PIK-93
Voxtalisib
WYE-125132
PI3Ka_4409
AKT inhibitor VIII
A-443654
Protein stability and degradation
RTK signaling
SU11274
Cediranib
Alectinib
NVP-TAE684
Unclassified
Elephantin
Fludarabine
Acetalax
WNT signaling
AZD7969
CHIR-99021
AZ6102
Effect size TP53 CFEs
Effect size TP53ness

Supplementary Figure 8. Pathway-wise effects of *TP53* mutations or phenocopies on drug sensitivity of cancer cell lines.

a) Drugs tested against *TP53* status in a pan-cancer manner. Each dot represents a drug. Each panel represents a drug targeted pathway. X axis represents the p-valueresultant after testing IC50 values against *TP53* phenocopy labels. Y axis represents the same but using *TP53* mutational status (CFEs). Hits under a 25% FDR are labelled. Intercept is shown as a line.

b) Drugs tested against *TP53* status in a pan-cancer manner. Each dot represents a drug. Each panel represents a drug targeted pathway. X axis represents the effect size (Cohen's d) resultant after testing IC50 values against *TP53* phenocopy labels.Y axis represents the same but using *TP53* mutational status (CFEs). Hits under a 25% FDR are labelled. Intercept is shown as a line.

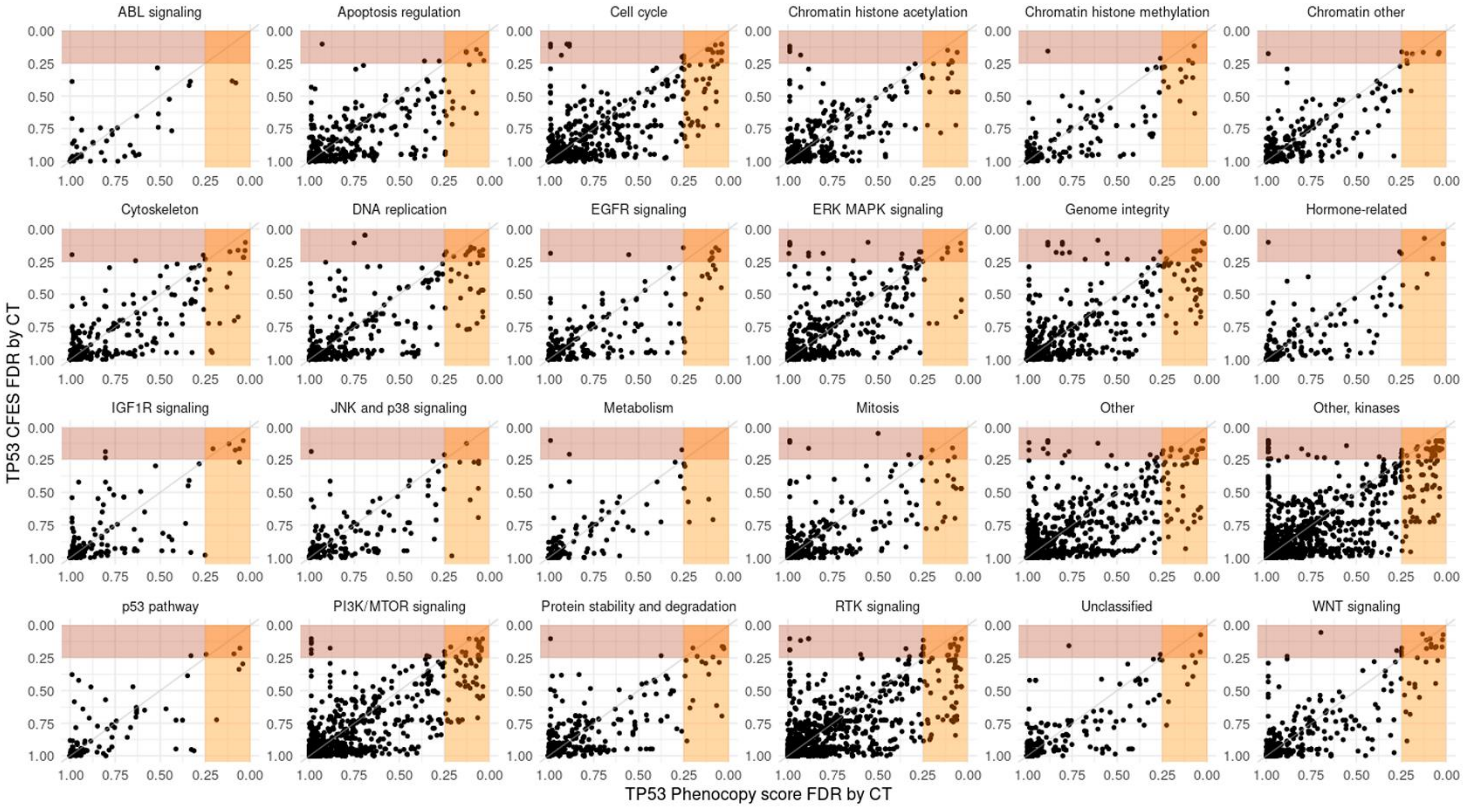

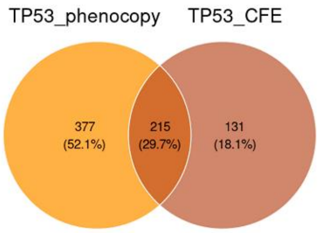

Supplementary Figure 9. Cancer type specific effect of TP53 phenocopies or mutations in drug testing

Drugs tested against *TP53* status by cancer type. Each dot represents a drug tested in a given cancer type. Each panel represents a drug targeted pathway. X axis represents the FDR resultant after testing log IC50 values against *TP53* phenocopy labels. The Y axis represents the same but using *TP53* mutational status (CFEs). Orange-colored area represents FDR<25% for *TP53* phenocopy hits, while red colores area represents the same for *TP53* CFEs. Venn diagram shows the number of hits found using each of the *TP53* labels: orange for FDR < 25% hits using *TP53* phenocopy labels and red for FDR < 25% using *TP53* mutational status (CFEs).

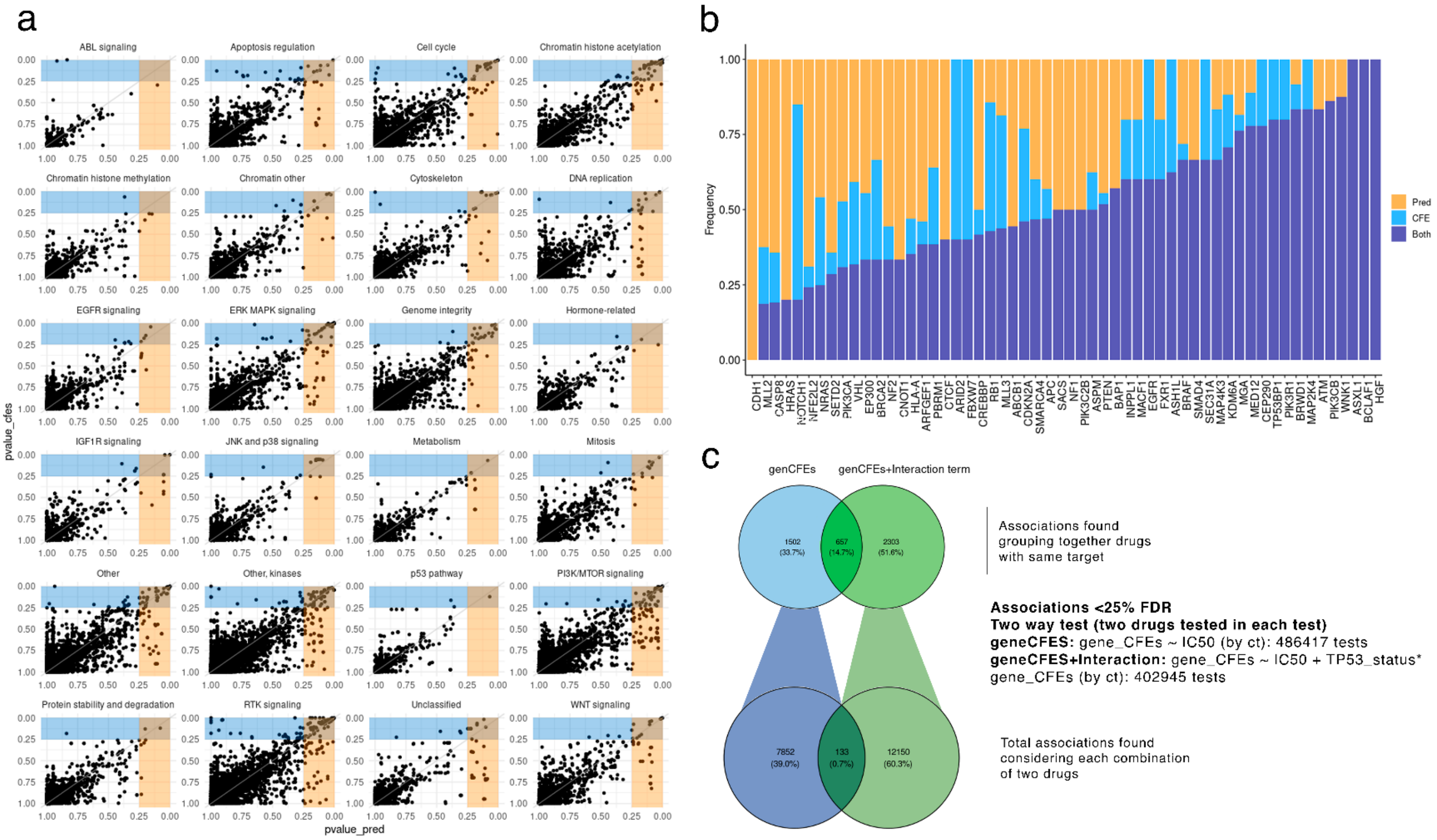

Supplementary Figure 10. Associations between mutation status of various genes and the sensitivity to various drugs can be modulated by the *TP53* functional status.

a) Associations found by pathway when testing GDSC drugs against gene CFEs using *TP53* status as interaction term. Each panel represents a pathway. Each dot represents associations of gene status with a drug targeting the given pathway, by cancer type. The Y axis represents the association´s FDR using *TP53* mutational status (CFEs). X axis represents the same using TP53 phenocopy labels. Yellow area contains associations with FDR<0.25 for *TP53* phenocopy labels. Blue area illustrates the same but for *TP53* CFEs.

b) TP53 phenocopy score recovery over *TP53* mutational CFEs by gene. Y axis represents the frequency of hits found by cancer type when a regression IC50 isfitted against gene CFEs using as interaction: *TP53* phenocopy score labels, *TP53* mutational status (CFE) or both. X axis represents a selection of top geneswith the highest frequency of associations.

c) Number of hits (FDR<25%) found just using gene CFEs versus using gene CFEsplus TP53 phenocopy score status as interaction term in a regression with GDSCdrugs IC50 values by cancer type. Top Venn diagram represents associations merged (hits with the same molecular target are counted together) by targeted genes - gene CFEs. Bottom Venn diagram represents all associations of drug_A-drug_B-gene CFEs.

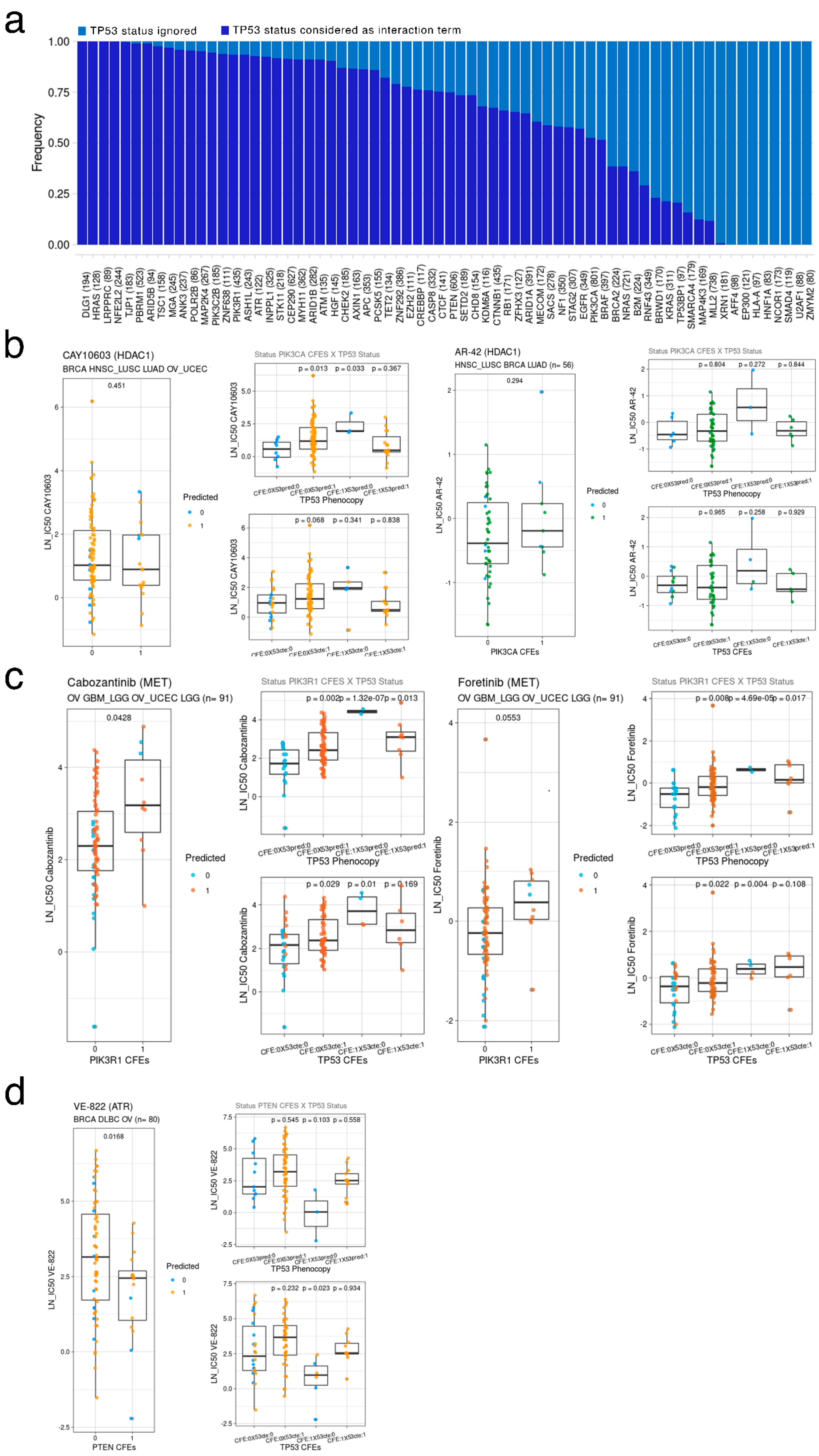
a
TP53 status ignored
TP53 status considered as interaction term
Frequency
1.00
0.75
0.50
0.25
0.00
DLG1 (194) HRAS (128) LRPPRC (89) NFE2L2 (244) TJP1 (183) PBRM1 (523) ARID5B (94) TSC1 (158) MGA (245) ANK3 (237) POLR2B (86) MAP2K4 (267) PIK3C2B (185) ZNF638 (111) PIK3R1 (435) ASH1L (243) ATR (122) INPPL1 (325) STK11 (218) CEP290 (627) MYH11 (362) ARID1B (282) ATM (135) HGF (145) CHEK2 (185) AXIN1 (163) APC (353) PCSK5 (155) TET2 (134) ZNF292 (386) EZH2 (111) CREBBP (117) CASP8 (332) CTCF (141) PTEN (606) SETD2 (189) CHD8 (154) KDM6A (116) CTNNB1 (435) RB1 (171) ZFHX3 (127) ARID1A (391) MECOM (172) SACS (278) NF1 (350) STAG2 (307) EGFR (349) PIK3CA (801) BRAF (397) BRCA2 (224) NRAS (721) B2M (224) RNF43 (349) BRWD1 (170) KRAS (311) TP53BP1 (97) SMARCA4 (179) MAP4K3 (169) MLL2 (738) XRN1 (181) AFF4 (98) EP300 (121) HLA-A (97) HNF1A (83) NCOR1 (173) SMAD4 (119) U2AF1 (88) ZMYM2 (80)
b
CAY10603 (HDAC1)
BRCA HNSC_LUSC LUAD OV_UCEC
0.451
LN_IC50 CAY10603
Predicted
0
1
Status PIK3CA CFES X TP53 Status
p = 0.013 p = 0.033 p = 0.367
CFE:0X53pred:0 CFE:0X53pred:1 CFE:1X53pred:0 CFE:1X53pred:1
TP53 Phenocopy
p = 0.068 p = 0.341 p = 0.838
CFE:0X53cte:0 CFE:0X53cte:1 CFE:1X53cte:0 CFE:1X53cte:1
AR-42 (HDAC1)
HNSC_LUSC BRCA LUAD (n= 56)
0.294
LN_IC50 AR-42
PIK3CA CFEs
Status PIK3CA CFES X TP53 Status
p = 0.804 p = 0.272 p = 0.844
TP53 Phenocopy
p = 0.965 p = 0.258 p = 0.929
TP53 CFEs
c
Cabozantinib (MET)
OV GBM_LGG OV_UCEC LGG (n= 91)
0.0428
LN_IC50 Cabozantinib
PIK3R1 CFEs
Status PIK3R1 CFES X TP53 Status
p = 0.002 p = 1.32e-07 p = 0.013
TP53 Phenocopy
p = 0.029 p = 0.01 p = 0.169
TP53 CFEs
Foretinib (MET)
OV GBM_LGG OV_UCEC LGG (n= 91)
0.0553
LN_IC50 Foretinib
PIK3R1 CFEs
Status PIK3R1 CFES X TP53 Status
p = 0.008 p = 4.69e-05 p = 0.017
TP53 Phenocopy
p = 0.022 p = 0.004 p = 0.108
TP53 CFEs
d
VE-822 (ATR)
BRCA DLBC OV (n= 80)
0.0168
LN_IC50 VE-822
PTEN CFEs
Status PTEN CFES X TP53 Status
p = 0.545 p = 0.103 p = 0.558
TP53 Phenocopy
p = 0.232 p = 0.023 p = 0.934
TP53 CFEs

Supplementary Figure 11. Additional analyses of TP53 status modulating associations between driver mutations and drugs.

- **a)** By gene improvement of TP53 phenocopy score as interaction term. We fitted a regression by cancer type to each drug against: gene CFEs labels ignoring *TP53* and gene CFEs labels plus TP53 phenocopy score labels as interaction. The Y axis represents the frequency of hits with an FDR < 0.25. X axis represents the different genes and the number of associations found significant by cancer type (using any label). Top genes with more than 70 associations are shown.
- **b)** Left (green-blue): validating the associations of AR-42 sensitivity to *PIK3CA* mutation status, when stratifying by TP53 status, using the PRISM screening data set. Right (Red-blue): association of another HDAC1i (Belinostat) to *PIK3CA* gene driver mutations (CFEs) when stratifying by TP53 status. Structure of the plots follow Fig. 6 B, C structure. Both associations were found by the two-way test (see Methods, hit PRISM: AR-42 & Belinostat vs *PIK3CA* CFEs, hit GDSC: CAY10603 & AR-42 vs *PIK3CA* CFEs). Only one drug of the two-way association is plotted.
- **c)** METi associations to *PIK3R1* gene CFEs when considering TP53 status. Left: association of Cabozantinib with *PIK3R1* status. Right: association of Foretinib with *PIK3R1* status. Organization is as in the Figure 5.
- **d)** VE-822 associations to PTEN driver mutations (CFEs) when considering TP53 status (two-way GDSC hit: VE-822 & VE-821 vs PTEN CFEs). Just one drug of the two-way association is plotted. Organization of the plots follow Figure 5.
