# Supplementary material for: Prevalence, causes and impact of TP53-loss phenocopying events in human tumors
Source: BMC Biol. 2023 Apr 24;21:92. doi: 10.1186/s12915-023-01595-1 (PMC10127307; doi:10.1186/s12915-023-01595-1)
Supplement: Supplementary file 2 — Additional file 2. [file 12915_2023_1595_MOESM2_ESM.pdf]

Supplementary Text 1.

CCR4-NOT is a transcription complex (CNOT), composed of 11 subunits, that plays an important role in multiple functions in terms of regulating translation, mRNA stability, and RNA polymerase I and II transcriptions [1,2]. CNOT2, one of the CCR4-NOT subunits, plays a critical role in deadenylase activity and the structural integrity of the complex [3] among other functions. An increasing number of studies have suggested CNOT2 role in tumor progression, such as in metastasis, proliferation and angiogenesis [4,5]. CNOT2 depletion and CCR4-NOT disruption have been linked to an apoptotic response via MID1IP1 and increased p53 activity [4, 6]. *CNOT2* has been reported to be among the top 5 amplified genes in chromosome 12, together with *MDM2* [7]. Its overexpression has been demonstrated in several cancer types such as pancreas, prostate, liver, urinary, ovarian and breast [5]. Experiments inducing CNOT2 overexpression led to increased p21 and p53 expression, decreased apoptosis and decreased TNF-related apoptosis-inducing ligand (TRAIL) sensitivity [6, 7].

Supplementary Text 2.

In BLCA, co-amplifications are associated with a higher TP53 phenocopy score, and are more frequent than *MDM2*-only amplifications (21 out of 32 are co-amplifications, Supp Fig. 6a, e). In BRCA, we found almost exclusively *MDM2-CNOT2* co-amplifications and no *MDM2*-only amplifications. In STAD co-amplifications of *MDM2* and *CNOT2* are more frequent (10 out of 13) than of *MDM2* solely. Just GBM was found to rely more on *MDM2*-only amplifications (8 out of 14, Supp Fig. 6e). Only 3 tumor samples were *CNOT2*-amplified but *MDM2*-non-amplified (all 3 having a TP53 phenocopy score lower than 0.5, Supp Fig. 6e). No cancer type relied on *CNOT2*-only amplifications

Supplementary Text References

1. Chekulaeva M, Mathys H, Zipprich JT, Attig J, Colic M, Parker R, et al. miRNA repression involves GW182-mediated recruitment of CCR4-NOT through conserved W-containing motifs. Nat Struct Mol Biol. 2011;18:1218–26.

2 Russell P, Benson JD, Denis CL. Characterization of mutations in NOT2 indicates that it plays an important role in maintaining the integrity of the CCR4-NOT complex. J Mol Biol. 2002;322:27–39.

3. Ito K, Inoue T, Yokoyama K, Morita M, Suzuki T, Yamamoto T. CNOT2 depletion disrupts and inhibits the CCR4-NOT deadenylase complex and induces apoptotic cell death. Genes Cells. 2011;16:368–79.

4. Sohn EJ, Jung D-B, Lee H, Han I, Lee J, Lee H, et al. CNOT2 promotes proliferation and angiogenesis via VEGF signaling in MDA-MB-231 breast cancer cells. Cancer Lett. 2018;412:88–98.

5. Jung JH, Lee D, Ko HM, Jang H-J. Inhibition of CNOT2 Induces Apoptosis via MID1IP1 in Colorectal Cancer Cells by Activating p53. Biomolecules. 2021;11.

6. Persson F, Olofsson A, Sjögren H, Chebbo N, Nilsson B, Stenman G, et al. Characterization of the 12q amplicons by high-resolution, oligonucleotide array CGH and expression analyses of a novel liposarcoma cell line. Cancer Lett. 2008;260:37–47.

7. Kim E-O, Kang S-E, Choi M, Rhee K-J, Yun M. CCR4-NOT transcription complex subunit 2 regulates TRAIL sensitivity in non-small-cell lung cancer cells via the STAT3 pathway. Int J Mol Med. 2020;45:324–32.
